# Supplementary material for: Surfactants or scaffolds? RNAs of varying lengths control the thermodynamic stability of condensates differently
Source: Biophys J. 2023 Mar 6;122(14):2973–87. doi: 10.1016/j.bpj.2023.03.006 (PMC10398262; doi:10.1016/j.bpj.2023.03.006)
Supplement: Document S1. Figures S1 and S2 [file mmc1.pdf]

Biophysical Journal, Volume *122*

Supplemental information

Surfactants or scaffolds? RNAs of varying lengths control the thermodynamic stability of condensates differently

Ignacio Sanchez-Burgos, Lara Herriott, Rosana Collepardo-Guevara, and Jorge R. Espinosa

Supplementary Material: Surfactants or scaffolds? RNAs of different lengths exhibit heterogeneous distributions and play diverse roles in RNA-protein condensates

Ignacio Sanchez-Burgos[‡,1], Lara Herriott[‡,1], Rosana Collepardo-Guevara[2,3,*] and Jorge R. Espinosa[1,4,*]
[1] Maxwell Centre, Cavendish Laboratory, Department of Physics, University of Cambridge, J J Thomson Avenue, Cambridge CB3 0HE, United Kingdom.
[2] Yusuf Hamied Department of Chemistry, University of Cambridge, Lensfield Road, Cambridge CB2 1EW, United Kingdom
[3] Department of Genetics, University of Cambridge, Cambridge, CB2 3EH
[4] Departament of Chemical Physics, Faculty of Chemical Sciences, Universidad Complutense de Madrid, 28040 Madrid, Spain.
*‡ = These authors contributed equally to this work, * = To whom correspondence should be sent.*
(Dated: 2nd March 2023)

SI. MODELS AND SIMULATION DETAILS

A. Mpipi Model

For our simulations of *fused in sarcoma* (FUS) (see sequence below) and PR_{25} with polyU RNA, we employ the high-resolution sequence-dependent coarse-grained model Mpipi [1], which describes almost quantitatively the temperature-dependent LLPS phase behaviour of different protein condensates such as that of FUS. In addition, this model correctly predicts the multiphase behaviour of the polyR/polyK/polyU system, and recapitulates experimental LLPS trends for sequence mutations on FUS, DDX4 NTD and LAF-1 RRG domain variants [1]. Within this force field, electrostatic interactions are modelled through a Coulombic term with Debye–Huckel electrostatic screening [2], given by the sum over all particle-particle (i, j) interactions as:

$$E_{elec} = \sum_{i,j} \frac{q_i q_j}{4\pi\epsilon_r\epsilon_0 r_{ij}} exp(-\kappa r_{ij}) \tag{S1}$$

where q is the charge (being -0.75e for the different nucleotides: A, C, G, U; and +0.75e for amino acids such as R and K, +0.375e for H, and -0.75e for D and E residues), $\epsilon_r = 80$ is the relative dielectric constant of water, ϵ_0 is the electric constant, $\kappa^{-1} = 795$ pm is the Debye screening length, and r_{ij} is the distance separating particles i and j. For these interactions, a Coulomb cut-off of 3.5 nm is employed. The rest of non-bonded interactions (i.e., hydrophobic, cation-π or π-π) between distinct protein/RNA beads are modelled via the Wang–Frenkel potential [3]:

$$E_{WF} = \sum_{i,j} \epsilon_{ij}\alpha_{ij} \left[\left(\frac{\sigma_{ij}}{r_{ij}}\right)^{2\mu_{ij}} - 1\right] \left[\left(\frac{\sigma_{ij}}{r_{ij}}\right)^{2\mu_{ij}} - 1\right]^{2\nu_{ij}} \tag{S2}$$

where

$$\alpha_{ij} = 2\nu_{ij} \left(\frac{R_{ij}}{\sigma_{ij}}\right)^{2\mu_{ij}} \left[\frac{2\nu_{ij}+1}{2\nu_{ij}\left[\left(\frac{R_{ij}}{\sigma_{ij}}\right)^{2\mu_{ij}} - 1\right]}\right]^{2\nu_{ij}+1} \tag{S3}$$

representing σ_{ij} the molecular diameter of each residue/nucleotide and ϵ_{ij} the interaction strength between distinct amino acids and nucleotides (i and j). While σ_{ij}, ϵ_{ij} and μ_{ij} are parameters specified for each pair of interactions (see Ref. [1]), ν_{ij} and R_{ij} are constant model parameters set to $\nu_{ij} = 1$ and $R_{ij} = 3\sigma_{ij}$ for every interaction. Finally, bond energy is computed with an harmonic bond potential of the following form:

$$E_{bond} = \sum_{b} \frac{1}{2} k(r_b - r_0) \tag{S4}$$

where b is the total number of bonds, r_b is the bond distance, k=8.03 $Jmol^{-1}pm^{-2}$ is the spring constant and r_0 is the bond reference position, set to 381 pm and 500 pm for protein and RNA bonds respectively. For further details on this force field and the full list of the model parameters please see Ref. [1].

B. FUS Sequence and PDB of the structured domains

Full-FUS sequence

MASNDYTQQATQSYGAYPTQPGQGYSQQSSQPYGQQSYSGYSQSTDTSGYGQSSYSSYGQSQNTGYGTQSTPQGYGSTGGYGSS
QSSQSSYGQQSSYPGYGQQPAPSSTSGSYGSSSQSSSYGQPQSGSYSQQPSYGGQQQSYGQQQSYNPPQGYGQQNQYNSSSGGGG
GGGGGGNYGQDQSSMSSGGGSGGGYGNQDQSGGGGSGGYGQQDRGGRGRGGSGGGGGGGGGGYNRSSGGYEPRGRGGGRG
GRGGMGGSDRGGFNKFGGPRDQGSRHDSEQDNSDNNTIFVQGLGENVTIESVADYFKQIGIIKTNKKTGQPMINLYTDRETGKL
KGEATVSFDDPPSAKAAIDWFDGKEFSGNPIKVSFATRRADFNRGGGNGRGGRGRGGPMGRGGYGGGGSGGGGRGGFPSGGG
GGGGQQRAGDWKCPNPTCENMNFSWRNECNQCKAPKPDGPGGGPGGSHMGGNYGDDRRGGRGGYDRGGYRGRGGDRGGF
RGGRGGGDRGGFGPGKMDSRGEHRQDRRERPY

The following Protein Data Bank (PDB) codes were used to build the globular structured domains of FUS (residues from 285–371 (PDB code: 2LCW) and from 422–453 (PDB code: 6G99)).

C. Colloidal patchy particle model for protein/RNA phase-separation

For the minimal coarse-grained simulations shown in Fig. 4 of the main text, we employ a patchy particle model [4–7] in which proteins are described by a pseudo hard-sphere (PHS) potential [8] that accounts for their excluded volume:

$$E_{PHS} = \sum_{i<j} \begin{cases} \lambda_r (\frac{\lambda_r}{\lambda_a})^{\lambda_a} \varepsilon_R \left[(\frac{\sigma}{r_{ij}})^{\lambda_r} - (\frac{\sigma}{r_{ij}})^{\lambda_a} \right] + \varepsilon_R; & \text{if } r < (\frac{\lambda_r}{\lambda_a})\sigma \\ 0; & \text{if } r \geq (\frac{\lambda_r}{\lambda_a})\sigma \end{cases} . \tag{S5}$$

where λ_a =49 and λ_r=50 are the exponents of the attractive and repulsive terms respectively, and ε_R accounts for the energy shift of the pseudo hard-sphere interaction. On top of this, we add a continuous square-well (CSW) potential for modeling the different protein binding sites, therefore mimicking protein multivalency:

$$E_{CSW} = \sum_{i<j} -\frac{1}{2}\epsilon_{CSW} \left[1 - tanh(\frac{r_{ij} - r_w}{\alpha}) \right] \tag{S6}$$

where ϵ_{CSW} is the depth of the potential energy well, r_w the radius of the attractive well, and α controls the steepness of the well. We choose $\alpha = 0.005\sigma$ and $r_w = 0.12\sigma$ so that each binding site can only interact with another single one. RNA-protein interactions are modeled with a standard Lennard-Jones (LJ) potential [9]:

$$E_{LJ} = \sum_{i<j} 4\epsilon_{LJ} \left[\left(\frac{\sigma}{r_{ij}} \right)^{12} - \left(\frac{\sigma}{r_{ij}} \right)^{6} \right] \tag{S7}$$

where ϵ_{LJ} measures the depth of potential and σ the excluded volume between proteins and RNA. The Lennard-Jones potential is employed between protein cores (not binding sites) and RNA beads, while RNA-RNA interactions are modelled via the PHS potential [8], with a repulsive Yukawa potential on top of the PHS one:

$$E_{Yukawa} = \sum_{i<j} A \frac{e^{-\kappa r_{ij}}}{r_{ij}} \tag{S8}$$

where we set A at 0.42 kcal·Å / mol and κ at 2.57 Å^{-1}. In this way, we model RNA as a self-repulsive polymer of bonded repulsive spheres, and protein-RNA interactions via sites that are not the protein-protein binding sites. Hence, if one protein is bonded to RNA, it can still bind to other proteins. In our model, the mass of each patch is 5% of the central PHS particle mass, which is set to 3.32x10^{-26}kg, despite this choice being irrelevant for equilibrium simulations. This 5% ratio fixes the moment of inertia of the patchy particles (our minimal coarse-grained proteins). The molecular diameter of the proteins, both scaffold and cognate proteins, as well as the RNA beads is $\sigma = 0.3405$

nm, and the value of ε_R/k_B is 119.81K. With this model, we express magnitudes in reduced units: reduced temperature is defined as $T^* = k_BT/\epsilon_{CSW}$, reduced density as $\rho^* = (N/V)\sigma^3$, reduced pressure as $p^* = p\sigma^3/(k_BT)$, and reduced time as $\sqrt{\sigma^2 m/(k_BT)}$. In order to keep the PHS interaction as similar as possible to a pure HS interaction, we fix k_BT/ε_R at a value of 1.5 as suggested in Ref. [8] (fixing $T = 179.71$K). We then control the effective strength of the binding protein attraction by varying ϵ_{CSW} such that the reduced temperature, $T^* = k_BT/\epsilon_{CSW}$, is of the order of $\mathcal{O}(0.1)$. The cut-off distance for the interactions in this model are 1.17σ for both PHS and CSW potentials and 5σ for the LJ interactions. The ϵ_{LJ}/k_B for LJ interactions is set to 152.5K.

This model has been proven to qualitatively reproduce the effect of protein valency in LLPS [5], the enhancement of RNA-mediated LLPS in RNA-binding proteins [10] or the multilayer organization [6] and condensate size conservation in scaffold-client mixtures [7].

D. Simulation details

Our Direct Coexistence simulations [11, 12] are performed in the NVT ensemble (i.e. constant number of particles (N), volume (V) and temperature (T)), for which we use a Nosé–Hoover thermostat [13, 14] with a relaxation time of 5 ps for the Mpipi model simulations and 0.074 in reduced units for the patchy particle simulations. Since all our potentials are continuous and differentiable, we perform all our simulations using the LAMMPS Molecular Dynamics package [15]. Periodic boundary conditions are used in the three directions of space. The timestep chosen for the Verlet integration of the equations of motion is 10 fs for the Mpipi model and 3.7x10^{-4} in reduced time units for the patchy particle model. For the bulk simulations with the Mpipi model, we employ the NpT ensemble, controlling the pressure (p) with the Nosé–Hoover barostat [16] using a relaxation time of 10 ps. All our simulations were equilibrated for at least 50 ns, ensuring that quantities such as the potential energy of the system and the condensate density remain constant over time. Production runs generally last from 200 ns up to few microseconds depending on the system and the property of interest, being more computationally demanding those simulations aiming to elucidate the condensate architecture (as shown in Figs. 3 (main text) and S1).

PR_{25}-polyU simulations were performed with 96 repeats of the protein and varying amounts of RNA, ranging from 1600 to 4800 nucleotides in total, and split into different chains to achieve the desired mass ratio and polyU strand length. For FUS-polyU simulations, we make use of 48 protein replicas and 400 to 1600 nucleotides in total forming RNA chains of different lengths. In the minimal model simulations we made use of 1000 coarse-grained proteins along with 100 to 600 RNA beads in total split into chains of different lengths as indicated in the main text.

SII. COMPUTING PHASE DIAGRAMS VIA DIRECT COEXISTENCE

To calculate the coexisting densities of the phase diagrams shown in Fig. 1b and 4b-c of the main text, we employ the Direct Coexistence method [11, 12, 17]. Within this scheme, the two coexisting phases are simulated by preparing periodically extended slabs of the two phases, the condensed and the diluted one, in the same simulation box. We use an implicit solvent model; accordingly, the diluted phase (protein-poor liquid phase) and the condensed phase (protein-rich liquid phase) are effectively a vapour and a liquid phase, respectively. Once our DC simulations have reached equilibrium, we compute the density profile along the long axis of the box, and thus, we extract the density of the two coexisting phases (as shown in Fig. 1(b) and 3(a-e) of the main text and the Supporting Material of Ref. [10]). From the plateau of the condensed phase and the diluted one, we measure the density (avoiding the interfaces between both phases). To estimate the critical point of the phase diagrams, we use the universal scaling law of coexistence densities near a critical point [18], and the law of rectilinear diameters [19]:

$$\big(\rho_l(T) - \rho_v(T)\big)^{3.06} = d\left(1 - \frac{T}{T_c}\right) \tag{S9}$$

and

$$(\rho_l(T) + \rho_v(T))/2 = \rho_c + s_2(T_c - T) \tag{S10}$$

where ρ_l and ρ_v refer to the coexisting densities of the condensed and diluted phases respectively, while ρ_c is the critical density, T_c is the critical temperature, and d and s_2 are fitting parameters. On the other hand, the methodology to estimate critical temperatures via NpT bulk simulations is provided in Section II of the main text, and illustrated in Fig. 1b.

SIII. COMPUTING THE INTERFACIAL FREE ENERGY FROM DIRECT COEXISTENCE SIMULATIONS

From Direct Coexistence simulations, we can obtain the interfacial free energy (γ) of the condensates by using the following expression:

$$\gamma = \int_{-\infty}^{\infty} [p_N(x) - p_T(x)]\, dx = \frac{L_x}{2N}(\overline{p}_N - \overline{p}_T) \tag{S11}$$

where L_x corresponds to the long side of the simulation box (perpendicular to the slab interfaces), N is the number of droplets in the system, and p_N and p_T are the normal and tangential components of the pressure tensor with respect to the interfaces of the system (note that the tangential component must be averaged over the two tangential directions).

SIV. SIMULATION CONVERGENCE

To ensure that our simulations reach convergence, we estimate from our bulk NpT simulations (presented in Fig. 1 and 2 of the main text) the approximate relaxation timescale for the systems to equilibrate. This can be easily approximated from the time that it takes the centre-of-mass particle of the largest biomolecule in the system to diffuse at least 1 or 2 times its average (protein or RNA) radius of gyration (as discussed in Refs. [20–22]). Once simulated the timescale needed to diffuse such distance (typically between 3 to 5 microseconds), we compute density profiles of the system over time, and we average the profiles from independent blocks to determine the equilibrium condensate structural arrangement (as shown in Fig. 3). Importantly, we note that the impact of a modest equilibration in systems employed to determine the critical temperature can be less dramatic than that required for characterizing the condensate architecture as shown in Fig. 3 of the main text, and Fig S1.

SV. EFFECT OF TEMPERATURE ON THE RNA-PROTEIN CONDENSATE STRUCTURAL ARRANGEMENT

In Fig. 3 of the main text, we present density profiles for FUS-RNA and PR_{25}-RNA condensates at T=0.98T$_c$. In order to assess the effect of temperature on the observed condensate architecture, in Fig. S1 we show density profiles for the same condensates but at T/T$_c$ 0.95 and 0.85 to demonstrate the mild dependence of condensate organization on temperature. We observe that there is no significant variation with temperature in the local arrangement of proteins and RNA along the condensate, with the density profiles presented in Fig. S1 being very similar to those of Fig. 3 in the main text.

SVI. PROTEIN-RNA CONTACT FREQUENCY MAPS

As mentioned in the main text, the Mpipi model, despite being a residue-resolution coarse-grained model, it is able to qualitatively recapitulate the different contact frequencies that distinct protein domains can establish with RNA [1]. Here, following the methodology described in Refs. [21–23], we evaluate the contact frequency between polyU RNA strands and the different protein amino acids of both FUS and PR_{25} within the condensates. In Figure S2, we show the contact map frequency of the different residues in both FUS-RNA and PR_{25}-RNA condensates for the heterotypic protein-RNA interactions. This calculation has been performed at T=0.95T$_c$ and in the presence of both long (400-nt) and short (50-nt) RNA chains for both PR_{25}-RNA and FUS-RNA systems (i.e., same conditions of the results shown in Fig. S1 for T=0.95T$_c$). For the case of the PR_{25}-RNA system, the U/PR_{25} mass ratio was 1.6, while for the FUS-RNA condensate it was U/FUS mass ratio = 0.14. While in PR_{25}-RNA droplets protein-RNA

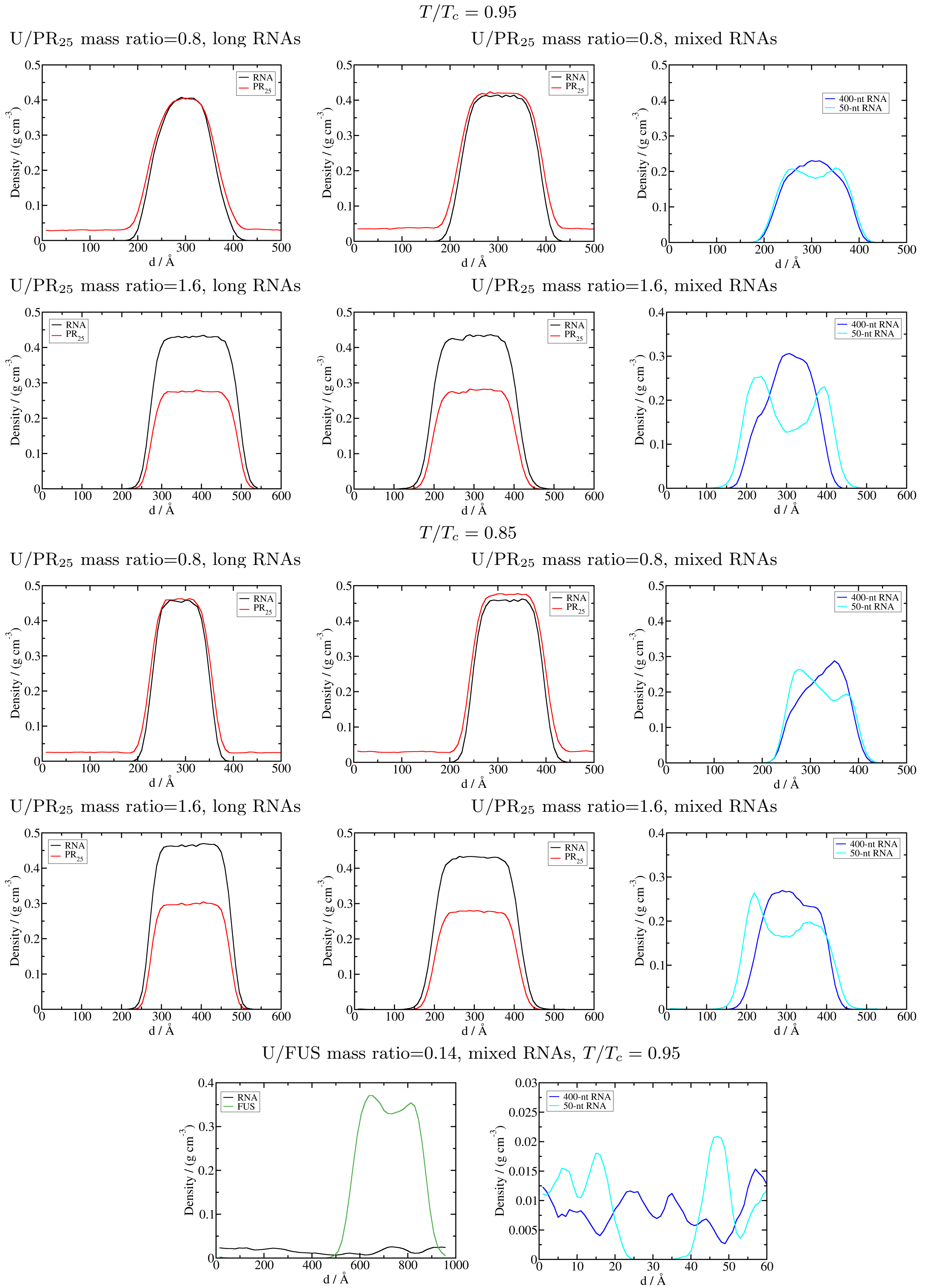

Figure S1: RNA-protein density profiles for the condensates shown in Fig. 3 of the main text and at the temperatures indicated in the different panels.

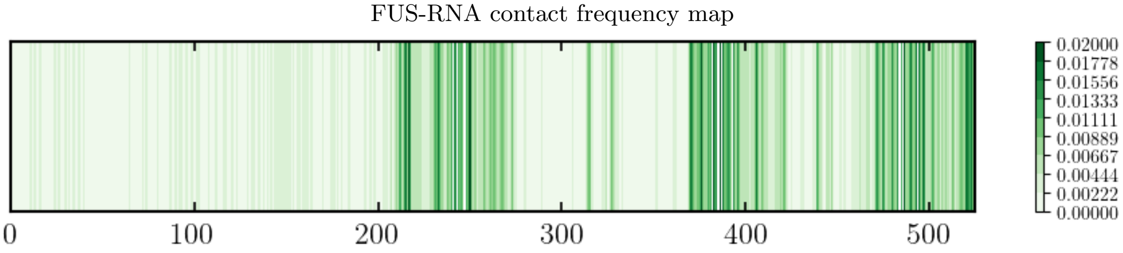

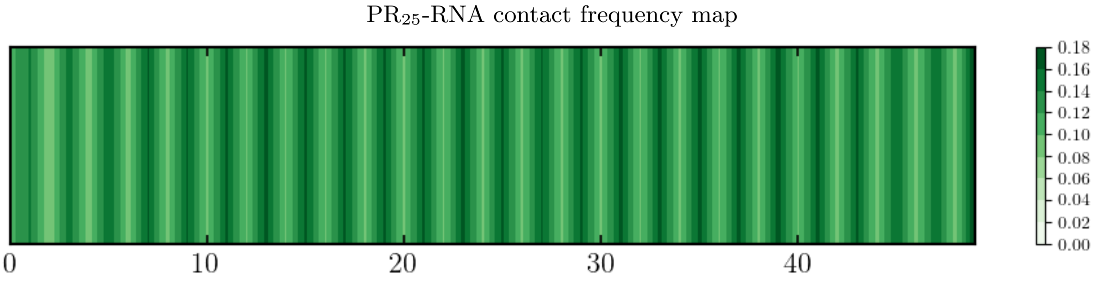

Figure S2: Contact probability per residue between polyU and the different amino acids composing FUS-RNA (Top panel) and PR_{25}-RNA (Bottom panel) condensates. The bar indicates the frequency of amino acid-U contacts (normalized by the averaged total number of protein-RNA contacts), with the darkest colours corresponding to a higher number of contacts. Calculations were performed at T=0.95T_c (T_c being the corresponding critical temperature of each system) and in the presence of both long (400-nt) and short (50-nt) RNA chains for both PR_{25}-RNA and FUS-RNA systems (as studied in Fig. S1). For the case of the PR_{25}-RNA system, we select a U/PR_{25} mass ratio of 1.6, while for the FUS-RNA a U/FUS mass ratio of 0.14.

interactions are quite homogeneous due to the dipeptide repeat distribution of the PR_{25} sequence, in FUS-RNA condensates, the domains which preferentially interact with polyU are the RNA-recognition motifs (from the 282nd to the 371st residue), and the three arginine-glycine rich regions (RGG1: 163 to 267; RGG2: 371 to 422; RGG3: 453 to 526), in qualitative agreement with experimental observations [24–26].

[1] A. J. Joseph, A. Reinhardt, A. Aguirre, P. Y. Chew, K. O. Russell, J. R. Espinosa, A. Garaizar, and R. Collepardo-Guevara, Nat. Comput. Sci. , in press (2021).
[2] P. Debye and E. Hückel, Physikalische Zeitschrift **24**, 185 (1923).
[3] X. Wang, S. Ramírez-Hinestrosa, J. Dobnikar, and D. Frenkel, Physical Chemistry Chemical Physics **22**, 10624 (2020).
[4] J. R. Espinosa, A. Garaizar, C. Vega, D. Frenkel, and R. Collepardo-Guevara, J. Chem. Phys **150**, 224510 (2019).
[5] J. R. Espinosa, J. A. Joseph, I. Sanchez-Burgos, A. Garaizar, D. Frenkel, and R. Collepardo-Guevara, Proceedings of the National Academy of Sciences (2020).
[6] I. Sanchez-Burgos, J. R. Espinosa, J. A. Joseph, and R. Collepardo-Guevara, Biomolecules **11**, 278 (2021).
[7] I. Sanchez-Burgos, J. A. Joseph, R. Collepardo-Guevara, and J. R. Espinosa, bioRxiv (2021).
[8] J. Jover, A. J. Haslam, A. Galindo, G. Jackson, and E. A. Müller, Journal of Chemical Physics **137** (2012), 10.1063/1.4754275.
[9] J. E. Jones, Proceedings of the Royal Society of London. Series A, Containing Papers of a Mathematical and Physical Character **106**, 441 (1924).
[10] J. A. Joseph, J. R. Espinosa, I. Sanchez-Burgos, A. Garaizar, D. Frenkel, and R. Collepardo-Guevara, Biophysical Journal **120**, 1219 (2021).
[11] A. J. Ladd and L. V. Woodcock, Chemical Physics Letters **51**, 155 (1977).
[12] R. García Fernández, J. L. F. Abascal, and C. Vega, The Journal of Chemical Physics **124**, 144506 (2006).
[13] S. Nosé, The Journal of Chemical Physics **81**, 511 (1984).

[14] W. G. Hoover, Phys. Rev. A **31**, 1695 (1985).
[15] S. Plimpton, Journal of Computational Physics **117**, 1 (1995).
[16] W. G. Hoover, Physical Review A **34**, 2499 (1986).
[17] J. R. Espinosa, E. Sanz, C. Valeriani, and C. Vega, Journal of Chemical Physics **139** (2013), 10.1063/1.4823499.
[18] J. S. Rowlinson and B. Widom, *Molecular theory of capillarity* (Courier Corporation, 2013).
[19] J. A. Zollweg and G. W. Mulholland, The Journal of Chemical Physics **57**, 1021 (1972).
[20] A. Garaizar, J. R. Espinosa, J. A. Joseph, and R. Collepardo-Guevara, Scientific reports **12**, 4390 (2022).
[21] A. R. Tejedor, I. Sanchez-Burgos, M. Estevez-Espinosa, A. Garaizar, R. Collepardo-Guevara, J. Ramirez, and J. R. Espinosa, Nature communications **13**, 5717 (2022).
[22] A. R. Tejedor, A. Garaizar, J. Ramírez, and J. R. Espinosa, Biophysical Journal **120**, 5169 (2021).
[23] S. Blazquez, I. Sanchez-Burgos, J. Ramirez, T. Higginbotham, M. M. Conde, R. Collepardo-Guevara, A. R. Tejedor, and J. R. Espinosa, bioRxiv , 2022 (2022).
[24] B. Portz, B. L. Lee, and J. Shorter, Trends in biochemical sciences (2021).
[25] J. Wang, J.-M. Choi, A. S. Holehouse, H. O. Lee, X. Zhang, M. Jahnel, S. Maharana, R. Lemaitre, A. Pozniakovsky, D. Drechsel, *et al.*, Cell **174**, 688 (2018).
[26] S. Maharana, J. Wang, D. K. Papadopoulos, D. Richter, A. Pozniakovsky, I. Poser, M. Bickle, S. Rizk, J. Guillén-Boixet, T. M. Franzmann, *et al.*, Science **360**, 918 (2018).
