# Supplementary material for: Surfactants or scaffolds? RNAs of varying lengths control the thermodynamic stability of condensates differently
Source: Biophys J. 2023 Mar 6;122(14):2973–87. doi: 10.1016/j.bpj.2023.03.006 (PMC10398262; doi:10.1016/j.bpj.2023.03.006)
Supplement: Document S2. Article plus supporting material [file mmc2.pdf]

Biophysical Journal
Article

Biophysical Society

Surfactants or scaffolds? RNAs of varying lengths control the thermodynamic stability of condensates differently

Ignacio Sanchez-Burgos,[1] Lara Herriott,[1] Rosana Collepardo-Guevara,[2,3,*] and Jorge R. Espinosa[1,4,*]
[1]Maxwell Centre, Cavendish Laboratory, Department of Physics, University of Cambridge, Cambridge, United Kingdom; [2]Yusuf Hamied Department of Chemistry, University of Cambridge, Cambridge, United Kingdom; [3]Department of Genetics, University of Cambridge, Cambridge, United Kingdom; and [4]Departament of Chemical Physics, Faculty of Chemical Sciences, Universidad Complutense de Madrid, Madrid, Spain

ABSTRACT Biomolecular condensates, thought to form via liquid-liquid phase separation of intracellular mixtures, are multicomponent systems that can include diverse types of proteins and RNAs. RNA is a critical modulator of RNA–protein condensate stability, as it induces an RNA concentration-dependent reentrant phase transition—increasing stability at low RNA concentrations and decreasing it at high concentrations. Beyond concentration, RNAs inside condensates can be heterogeneous in length, sequence, and structure. Here, we use multiscale simulations to understand how different RNA parameters interact with one another to modulate the properties of RNA–protein condensates. To do so, we perform residue/nucleotide resolution coarse-grained molecular dynamics simulations of multicomponent RNA–protein condensates containing RNAs of different lengths and concentrations, and either FUS or PR_{25} proteins. Our simulations reveal that RNA length regulates the reentrant phase behavior of RNA–protein condensates: increasing RNA length sensitively rises the maximum value that the critical temperature of the mixture reaches, and the maximum concentration of RNA that the condensate can incorporate before beginning to become unstable. Strikingly, RNAs of different lengths are organized heterogeneously inside condensates, which allows them to enhance condensate stability via two distinct mechanisms: shorter RNA chains accumulate at the condensate's surface acting as natural biomolecular surfactants, while longer RNA chains concentrate inside the core to saturate their bonds and enhance the density of molecular connections in the condensate. Using a patchy particle model, we additionally demonstrate that the combined impact of RNA length and concentration on condensate properties is dictated by the valency, binding affinity, and polymer length of the various biomolecules involved. Our results postulate that diversity on RNA parameters within condensates allows RNAs to increase condensate stability by fulfilling two different criteria: maximizing enthalpic gain and minimizing interfacial free energy; hence, RNA diversity should be considered when assessing the impact of RNA on biomolecular condensates regulation.

SIGNIFICANCE RNA is a critical modulator of the biophysical properties of biomolecular condensates, such as their stability and viscosity. Inside cells, a single condensate can contain RNAs of multiple different lengths at varying concentrations. Here, we take advantage of multiscale simulations to investigate the intramolecular organization and biophysical properties of RNA–protein condensates that contain RNAs of different lengths. Our simulations reveal that RNAs of different lengths are distributed heterogeneously inside condensates. Short RNAs accumulate preferentially at the condensate's interface, acting as natural surfactants, whereas long RNAs concentrate deep within the core, strengthening the overall connectivity of the condensed liquid network. Furthermore, we demonstrate that RNA length and concentration cooperate to fine-tune the RNA-driven reentrant phase behavior of RNA–protein condensates: long RNAs increase both the maximum concentration of RNA that condensates can incorporate before dissolving, and their range of stability.

INTRODUCTION

Intracellular organization represents a fundamental aspect of regulation with respect to both structure and function. While membrane-bound organelles are responsible for forming large, often permanent compartments within the

Submitted November 9, 2022, and accepted for publication March 2, 2023.
*Correspondence: rc597@cam.ac.uk or jr752@cam.ac.uk
Ignacio Sanchez-Burgos and Lara Herriott contributed equally to this work.
Editor: Alberto Perez.
https://doi.org/10.1016/j.bpj.2023.03.006
© 2023 Biophysical Society.
This is an open access article under the CC BY license (http://creativecommons.org/licenses/by/4.0/).

cell, a more dynamic compartmentalization can be also achieved through membraneless organelles (1). Membraneless organelles, also referred to as biomolecular condensates, possess the two key properties of intracellular compartments: the existence of a defined boundary between the compartment and its surroundings, and the ability of components to diffuse freely within the compartment (2,3). Biomolecular condensates are thought to form via liquid–liquid phase separation (LLPS) of intracellular mixtures (e.g., proteins, RNA, DNA, and chromatin). Thus, the boundary of condensates is not a traditional lipid membrane, but rather the liquid–liquid interface separating a condensed liquid from its surrounding cytoplasm or nucleoplasm. Since the discovery of P-granule condensates in 2009 (4), important examples of biomolecular condensates including the nucleolus (5,6), Cajal bodies (7,8), paraspeckles (9,10), stress granules (11,12), and chromatin (13,14) (a finding that drove the paradigm shift away from the once prominent theory of the 30-nm fiber (15–17)) have been exhaustively investigated.

RNA-binding proteins are common components of intracellular biomolecular condensates (18–22). Several features of RNA-binding proteins underpin their ability to form condensates that are sensitively regulated by RNA. For instance, RNA-binding proteins are multidomain multivalent molecules, many of which can establish sufficiently strong homotypic interactions to act as scaffolds in biomolecular condensates—e.g., the heterogeneous nuclear ribonucleoprotein 1 (hnRNPA1) (12,23), fused in sarcoma (FUS) (24–26), the GTPase-activating protein SH3 domain-binding protein 1 (G3BP1) (27–30) and the transactivation response DNA-binding protein 43 (TDP-43) (31–33). In addition, RNA-binding proteins can bind to RNA both specifically and promiscuously via their RNA recognition motifs (RRMs), positively charged domains, and intrinsically disordered regions (IDRs) with low-complexity amino acid sequences (34–36). Both in vitro and, more recently, in silico experiments have demonstrated the role of RNA as a critical regulator of RNA-protein condensates (37–44). The RNA–binding protein Whi3 has been shown to partition into different condensates depending on the secondary structure of the RNA to which it is bound (45). Proteins such as FUS remain soluble in the nucleus, where RNA concentration is high, but form aggregates in the cytoplasm where RNA concentration is lower (46). Such impact of RNA concentration on protein aggregation may be relevant to rationalize the presence of pathological FUS aggregates (characteristic of amyotrophic lateral sclerosis) in the cytoplasm of postmortem tissues (47) versus their absence from the nuclei (48). The pattern of low levels of RNA promoting condensation versus higher concentrations promoting dissolution is described as RNA-driven reentrant phase behavior (37,43,46) and is particularly important when considering the role of RNA in complex coacervation (44,49–51). Complex coacervation frequently enables the phase separation of so-called cognate proteins, which, unlike FUS, cannot sustain LLPS through protein–protein interactions alone, but instead rely on interactions with a partner biomolecule such as RNA (52). The 25-repeat proline-arginine peptide (PR_{25}) is a representative example of a protein that undergoes complex coacervation at physiological conditions driven mostly by electrostatic interactions with RNA (44,51).

RNA length has been shown to mediate condensate reentrant phase behavior (53). Specifically, transcriptional condensate formation and dissolution was shown to be regulated by both RNA length and concentration: short, nascent RNAs present at transcription initiation stimulate condensation, while longer nucleic acids resulting from transcriptional bursts promote dissolution (53). The nature of transcriptional bursting, with the total number of RNA molecules as well as their lengths increasing (54), means it is unclear whether the reentrant phase behavior of transcriptional condensates is a function of RNA length, concentration, or a combination of both. In vitro experimentation has proved valuable in demonstrating the various ways in which RNA regulates LLPS of RNA-binding proteins (18–22,37–41,46). Complementary, computational modeling and simulations can provide mechanistic insight into the experimental observations, and molecular detail regarding the condensate's thermodynamic, kinetic, and structural properties (55). Computer simulations can also elucidate condensate properties such as droplet surface tension, protein molecular contact maps, or protein/RNA/DNA conformational ensembles (56–61). Moreover, key features of LLPS, such as valency (62,63), topology (64,65), or binding affinity (66–68), can be precisely controlled in simulations. In that sense, simulations have proved useful for the study of biomolecular condensates at various levels of resolution: ranging from atomistic force fields to lattice-based physical models (16,51,69–72).

Here, we use molecular dynamics (MD) simulations, taking advantage of the benefits of computational modeling (56–61), to investigate in molecular detail the role of RNA length and concentration in the regulation of biomolecular condensates. Specifically, we aim to determine how RNA concentration and length cooperate or compete to affect the RNA-dependent reentrant phase behavior of RNA-binding proteins (18–20,37,38,44,46). We use our sequence-dependent Mpipi model (73), which predicts protein phase diagrams in quantitative agreement with experiments, to study the reentrant phase behavior of two archetypal proteins, FUS and PR_{25}, known to undergo LLPS either by homotypic interactions or complex coacervation, respectively (74). Furthermore, we investigate the effect of RNA length on condensate organization by simulating proteins with mixtures of RNA of different lengths. In particular, we aim to identify whether patterns similar to those previously described with colloidal scaffold–surfactant models (63) can also exist in RNA-protein systems. Finally, we show

that, even when we model biomolecules as simple patchy colloids (64), the strong influence of RNA length and concentration on the phase behavior of RNA–protein condensates can be captured, suggesting that such behavior is dictated by general physical parameters like the molecular valency, binding affinity, and polymer length.

MATERIALS AND METHODS

Since the formation of phase-separated condensates entails the collective interactions among thousands of different proteins and other biomolecules, the study of LLPS has benefited from the development and application of coarse-grained approaches, including mean field simulations (75–79), lattice-based models (80–83), minimal models (65,66), and residue-resolution simulations (57,58,67,84–88). In this work, we employ two protein/RNA coarse-grained models of different resolutions previously developed by us: 1) the residue/nucleotide resolution Mpipi force field for proteins and RNA (73) and 2) the MD-Patchy model in which whole proteins are represented as patchy particles, and RNA as self-avoiding flexible polymers (44,64,89).

Within the Mpipi force field (73), amino acids and RNA bases are represented by single beads with unique chemical identities (Fig. 1 *a*) in which hydrophobic, π–π, and cation–π interactions are modeled through a Wang-Frenkel (mid-range) potential (90), and electrostatic interactions via Yukawa/Debye–Hückel (long-range) potentials (84). Bonded interactions between consecutive residues within the same protein (or nucleotides within the same RNA strand) are described with a harmonic potential. Furthermore, within this model, intrinsically disordered regions of proteins and RNA strands are treated as fully flexible polymers, while globular domains are described as rigid bodies based on their corresponding crystal structures taken from the Protein Data Bank and adapted to the model resolution. The interactions between "buried" amino acids within globular domains are scaled down by 70% as done previously in (73,74). The solvent is modeled implicitly, and the screening effects of monovalent ions in solution at physiological concentrations (i.e., ~150 mM NaCl) are approximated by the Debye length of the Yukawa/Debye–Hückel potential. Because the Debye–Hückel potential invokes a mean field theory approximation, important effects such as ion condensation, ion correlations, ion heterogeneity, and specific ion binding are ignored. Additional effects that are not accounted for are the identity of the ions (e.g., Na^+, K^+ versus Mg^{2+}), and the entropic contributions of water (57,91). Thus, by using the Debye–Hückel potential, we assume that the effects of ions in solution can be approximated by the simple reduction of charge–charge interactions. Nonetheless, the approximation is exact in the low salt limit, and previous residue-resolution coarse-grained models invoking this approximation have been successfully employed to predict the phase diagrams and the single-molecule radii of gyration of proteins in quantitative agreement with experiments within the range of 100–150 mM NaCl (73,84,87). Regarding RNA–protein condensates, such implicit solvent coarse-grained models have been able to uncover molecular and thermodynamic mechanisms explaining their regulation (44,84,89,92). Like other molecular models with reduced physical details, these residue-resolution coarse-grained models are able to compensate for the innacuracies of the Debye–Hückel by using experimental and/or atomistic data in their parameterizations. Details on the force field parameters and simulation setups are provided in the supporting material.

In addition to the residue-resolution Mpipi model, we employ a minimal coarse-grained patchy model (MD-Patchy (64)) to elucidate whether the role of RNA length and concentration in RNA–protein condensates is determined by general molecular features such as valency, binding affinity, or the relative RNA/protein length. Within our patchy particle simulations, proteins are described as pseudo hard sphere (93) particles decorated with sticky patches, which account for the protein binding sites (modeled through square-well-like potentials (94)), and RNA strands are modeled as fully flexible self-avoiding polymers that can interact attractively with RNA-binding proteins via mid-range nonspecific interactions (89). Each RNA bead accounts for several nucleotides and has the same size as those of the proteins (89). Regarding the nonbonded potential, RNA–RNA interactions are described by a pseudo hard sphere potential in combination with a Yukawa/Debye–Hückel screened potential (for further details on the model potential and parameters see self-avoiding polymers trigger concentration-dependent reentrant phase behavior of colloidal patchy-particle condensates modulated by polymer length and supporting material) and an implicit solvent model. Accordingly, the diluted phase (i.e., the protein-poor liquid phase) and the condensed phase (i.e., the protein-rich liquid phase) are effectively a vapor and a liquid phase, respectively. Overall, the combination of both Mpipi and MD-Patchy models represents a multiscale approach for studying RNA–protein condensates given that the scale resolution of the patchy particles is approximately 2 orders of magnitude lower than that of the Mpipi residue-resolution model. Specifically, within the Mpipi model, FUS and PR_{25} are represented by ~500 and 50 amino acids, respectively. In comparison, only one bead is use for the whole proteins in the MD-Patchy simulations.

To determine the stability of RNA–protein condensates, we evaluate the phase diagrams (in the temperature-density plane) of the different systems by means of direct coexistence simulations (95,96). Within the direct coexistence approach (Fig. 1 *a*), the two coexisting phases of a given system are placed in the same simulation box. The simulation box used is rectangular, with an elongated side perpendicular to the interfaces—long enough to capture the bulk density of each phase—while the parallel sides are chosen

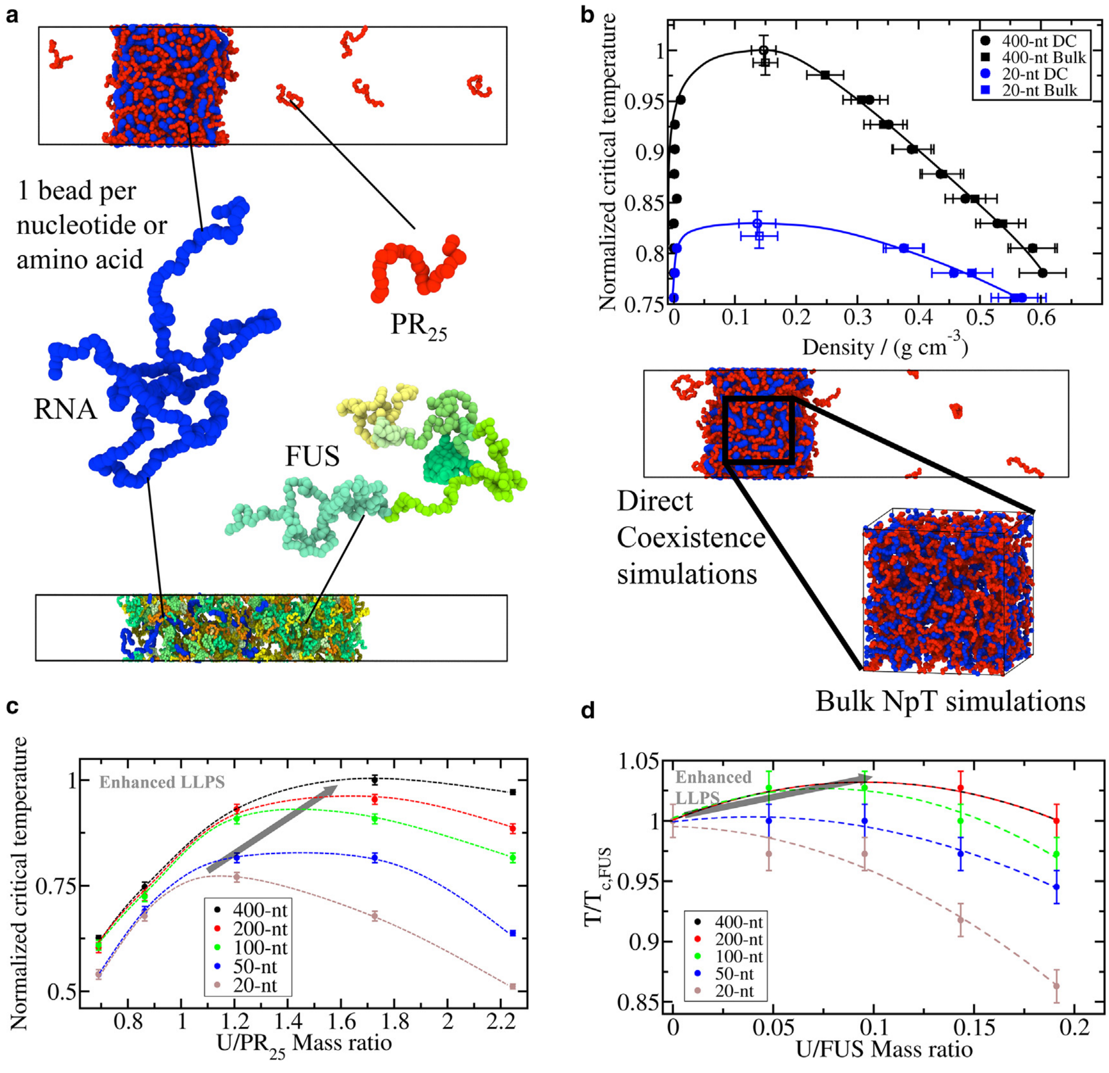

FIGURE 1 Reentrant phase behavior driven by RNA is regulated by both concentration and length. (*a*) Residue resolution coarse-grained simulations with the Mpipi model (73) to investigate phase separation of RNA–protein mixtures. Coarse-grained representation of (full sequence) FUS, PR_{25}, and a 400-nt polyU RNA strand using the Mpipi model (73) in which each amino acid or nucleotide is represented by a single bead. Please note that the size of the beads depicted in this panel has been conveniently rescaled for visualization purposes. In FUS protein, beads of different colors indicate different protein domains. Direct coexistence simulations of polyU-PR_{25} (*top*) and FUS-polyU (*bottom*) are also included. (*b*) Comparison of the predicted condensate densities as a function of temperature (renormalized by the highest critical temperature) for polyU–PR_{25} mixtures composed by polyU strands of 400 nt (*black symbols*) and 20 nt (*blue symbols*) using direct coexistence simulations (*solid circles*) and bulk *NpT* simulations (*solid squares*). The estimated critical temperature of each system by both ensembles is depicted by empty symbols of the corresponding shape and color. Snapshots of a direct coexistence simulation and a bulk *NpT* simulation are included to illustrate the analogy between both ensembles when describing the system condensed phase. Continuous lines represent the phase diagram coexistence lines. (*c*) Normalized critical temperature of polyU-PR_{25} mixtures as a function of the U/PR_{25} mass ratio for different polyU strand lengths as indicated in the legend. (*d*) Normalized critical temperature of FUS-polyU mixtures as a function of the U/FUS mass ratio for different polyU strand lengths as indicated in the legend. Dashed lines connecting the critical temperatures as a function of concentration are included as a visual guide. While in (*c*) all temperatures have been normalized by the highest T at which phase separation was observed ($T = 435$ K), in (*d*) all temperatures have been normalized by the critical temperature of pure FUS ($T_{c,FUS} = 365$ K). Please note that higher critical temperatures in our model correspond to higher driving forces to undergo LLPS (i.e., lower saturation concentration). To see this figure in color, go online.

such that proteins and RNA cannot interact with themselves across the periodic boundary conditions (43). We employ the canonical ensemble (constant number of molecules [N], system volume [(V)], and temperature [T], or NVT)). Once direct coexistence simulations reach equilibrium, we measure the coexisting densities of both the diluted and condensed phases along the long axis of the box, excluding the fluctuations at the interfaces and keeping the center of mass of the system fixed. By repeating this procedure at different temperatures—until we reach supercritical temperatures, i.e., where phase separation is no longer observed—we can evaluate phase diagrams (Fig. 1 *b*). Finally, to avoid

finite system-size effects close to the critical point, we estimate the critical temperature (T_c) and density (ρ_c) using the law of critical exponents and rectilinear diameters (97) (as shown in (64,89)). Fig 1 *a* shows a direct coexistence simulation with a system composed of PR_{25} and poly-uridine (polyU) RNA strands of 400 nucleotides (nt) and FUS with polyU strands of the same length at conditions in which both systems undergo LLPS.

To evaluate condensate densities while controlling accurately the concentration of individual components inside them, we perform additional simulations in the isothermal-isobaric ensemble (*NpT*). These *NpT* simulations allow us to fix the desired composition of a multicomponent condensate with ease, e.g., the RNA/protein proportion, which is difficult to do in the direct coexistence method. As shown in Fig. 1 *b*, *NpT* simulations provide a similar representation of the condensed phase as direct coexistence simulations—although avoiding the effects of interfaces. By fixing the system pressure to zero, and enabling the volume of the simulation box to isotropically fluctuate, we allow the condensed phase to equilibrate. Stable phase-separated condensates exhibit an equivalent coexistence density as that obtained from direct coexistence simulations, whereas unstable systems cannot sustain the condensed phase and tend toward infinitely dilute densities. Within the *NpT* ensemble, condensates are only stable at temperatures where the interactions between biomolecules are sufficiently strong to overcome the entropic cost of forming a percolating liquid network, without the need for pressure to be exerted on the simulation box. Finally, once the system densities are equilibrated, the critical temperature of each condensate can be estimated within the interval between the highest temperature at which the system is stable and the lowest temperature at which it is not. We note that the coexistence pressure from direct coexistence simulations deviates slightly from that imposed in *NpT* simulations (0 bar) at temperatures close to the critical one. However, to test the validity of determining critical temperatures through *NpT* simulations, we compare the condensate coexistence densities and critical temperatures obtained via *NpT* versus direct coexistence simulations for mixtures of polyU-PR_{25} with different strand lengths (Fig. 1 *b*). Based on a systematic difference of less than 2% in the predicted T_c, we conclude that the *NpT* approach to estimate critical temperatures is a reasonable method that allows us to have a better control of the RNA/protein ratio within condensates at all conditions.

RESULTS AND DISCUSSION

RNA length modulates the RNA concentration-dependent reentrant phase behavior of RNA–protein condensates

We first investigate how the RNA concentration-dependent reentrant phase behavior of RNA–protein condensates is influenced by the length of single-stranded RNA. For this, we perform direct coexistence and bulk *NpT* simulations to evaluate the critical temperature of each system (as detailed in materials and methods) using the residue/nucleotide resolution Mpipi force field (73), which has been shown to achieve quantitative agreement with experimental phase diagrams of RNA-binding proteins. We compare the behavior of two phase-separating RNA-binding proteins, FUS (526 residues, sequence in supporting material) and PR_{25}, for which phase behavior is modulated differently by RNA (44). FUS can form single-component condensates via homotypic interactions, which increase in stability at moderate RNA concentrations (37,43,46). PR_{25} is an arginine-rich peptide, which requires RNA to phase separate via heterotypic RNA–protein interactions at physiological conditions (44,51). For each case, we simulate solutions containing tens to hundreds of individual proteins in the presence of disordered single-stranded polyU RNA molecules with five different lengths: 20, 50, 100, 200, and 400 nt. For all RNA lengths, we test different polyU concentrations defined through the U/protein mass ratio, which allows us to quantify the total number of U nucleotides in the mixtures, regardless of whether they are assembled in longer or shorter polyU chains. Importantly, it has been shown that combining RNA-binding proteins and RNA at ratios resulting in electroneutral mixtures enhances the stability of RNA–protein condensates (42,43). Thus, here we explore a range of U/protein mass ratios that lie around the electroneutral point. We focus on single-stranded polyU RNA for simplicity and to follow previous studies on RNA-protein phase separation (18,37,74).

For the polyU-PR_{25} mixtures, the electroneutral point lies at the 1.21 U/protein mass ratio. Thus, we perform simulations for polyU-PR_{25} mixtures spanning the range between 0.78 and 2.24 U/PR_{25} mass ratios. For the electroneutral polyU-PR_{25} system, we first demonstrate that *NpT* simulations quantitatively reproduce the condensed phase coexistence densities found in phase diagrams constructed using standard *NVT* direct coexistence simulations (64,84). From a set of direct coexistence simulations at varying temperatures, we extract the phase diagrams for two polyU-PR_{25} mixtures containing RNA strands of varying lengths (20 and 400 nt) but keeping a constant U concentration (mass ratio of 1.21) regardless of RNA length (Fig. 1 *b*). Then, we simulate these systems in the *NpT* ensemble at zero pressure (using a cubic box). As shown in Fig. 1 *b*, this approach provides consistent condensate densities to those obtained via direct coexistence simulations (as long as the density of the dilute phase is very low). As our direct coexistence simulations indicate, approximating coexistence densities from *NpT* simulations is reasonable for most of the temperatures with densities of the dilute phase being of the order 1×10^{-3} g/cm^3 (please note that the solvent is implicitly considered within the force field). Since with *NpT* simulations the density of the dilute phase cannot be measured, the value for the critical temperature cannot be calculated using the law of critical exponents and rectilinear

diameters (97), as is the case in the direct coexistence simulations. However, an interval at which the critical temperature falls can be estimated. Fig. 1 *b* shows that the values of the critical temperatures estimated from bulk *NpT* simulations (as the mid temperature of the computed interval; described in materials and methods) lie within the uncertainty of the critical temperatures evaluated from direct coexistence simulations and the law of critical exponents and rectilinear diameters.

Having established that *NpT* simulations can provide robust estimates of the critical temperature, we move forward with the *NpT* ensemble to investigate the interplay between RNA concentration and the effects of RNA length on condensate stability. To consider the effect of RNA length independently, we keep the total amount of U nucleotides in the mixtures fixed, and assemble them in polyU chains of different lengths. When the U/PR_{25} mass ratio is kept constant, we observe a monotonic increase in the critical temperature of the condensates as the RNA length increases. For example, for polyU-PR_{25} mixtures at ratios satisfying the electroneutral point (i.e., 1.21 polyU/PR_{25} mass ratio), there is a 20% enhancement in the critical temperature when the RNA chain length increases from 20 to 400 nt (Fig. 1 *c*). Such behavior can be ascribed to the density of protein–RNA intermolecular contacts increasing significantly as the RNA lengthens, especially at strand lengths of tens to hundreds of nucleotides, as discussed in (44). Because PR_{25} must bind to RNA to form a condensed liquid network, adding covalent bonds within the RNA chains—for instance, by replacing many short strands with a longer one—increases the PR_{25}–RNA critical temperature by zipping together large chunks of RNA, which would otherwise be driven away by the dominant RNA–RNA electrostatic repulsion. When we next fix the RNA chain length, and investigate the impact of RNA concentration on the stability of polyU-PR_{25} condensates, for all the RNA lengths we study, we confirm that PR_{25} exhibits the well-known RNA concentration-dependent reentrant behavior of RNA-binding proteins discovered experimentally (22,37,46). That is, the stability of polyU-PR_{25} condensates—quantified by the values of the critical temperature—gradually increases as the RNA concentration goes from low to moderate (up to approximately the electroneutral point), then it reaches a maximum value, and finally decreases as the RNA concentration increases even further. Remarkably, looking at the combined effects of RNA length and concentration reveals that RNA length significantly modulates such reentrant behavior. Specifically, increasing RNA length sensitively raises the maximum value that the critical temperature of the mixture reaches (i.e., how much the condensate stability can be boosted by RNA), and the maximum concentration of U nucleotides that the condensate can incorporate before beginning to become unstable (i.e., when the RNA chains are longer, more nucleotides can form part of the condensate before it begins to dissolve). Thus, the most stable polyU-PR_{25} condensates are formed by the longest RNA chains we study, and, unexpectedly, contain U/PR_{25} concentrations above the electroneutral point (Fig. 1 *c*).

We next investigate whether such behavior also holds for proteins that are able to undergo phase separation on their own, i.e., via homotypic protein–protein interactions (65,98). For this, we focus on the protein FUS and test the impact of adding polyU of varying lengths and at different concentrations. Our simulations contain 48 replicas of FUS (full sequence, see supporting material) and polyU chains of 20, 50, 100, 200, or 400 nt in length. For all the different polyU lengths, we prepare mixtures at concentrations spanning the range of U/FUS mass ratio from 0 to 0.19. First, we calculate the critical temperature of pure FUS condensates, obtaining a value of $T_{c,FUS} = 365$ K. This temperature is in reasonable agreement (considering the coarse-grained nature of the model) with the experimental protein thermostability of FUS (~355 K (99)). Next, we look at mixtures containing RNA at the concentration corresponding to the electroneutral point (mass ratio of 0.049), and we confirm that performing *NpT* bulk simulations reproduces the length-dependent increase in critical temperature identified previously via direct coexistence simulations (44). Specifically, when we mix FUS with RNA of 100, 200, or 400 nt at the electroneutral ratio (0.049 U/FUS mass ratio), we see a marginal increase in critical temperature—3% with respect to the value for pure FUS system (Fig. 1 *d*)—in agreement with our previous direct coexistence results (44). Adding 20-nt polyU to FUS, the shortest polyU molecules we study, hinders phase separation, with the effect being amplified at higher polyU concentrations. This occurs because 20-nt polyU is too short to bridge FUS molecules and enhance the connectivity of the condensed liquid, as shown previously (43,44). For RNA lengths of 50, 100, 200, and 400 nt, the RNA concentration-dependent reentrant phase behavior of FUS is observed. That is, gradually increasing the concentration of polyU up to a given threshold (in our case a U/FUS mass ratio of around ~0.1) increases the critical temperature, while adding polyU at concentrations beyond such threshold reduces the critical temperature. The observation of peak critical temperatures for FUS condensates containing 50-nt polyU at polyU concentrations surpassing the electroneutral point (i.e., ~0.1), is consistent with in vitro studies (20). As in polyU–PR_{25} mixtures, we find that the maximum enhancement in phase separation occurs at higher polyU/protein mass ratios for longer-chain polyU systems. Overall, when polyU length is increased from 20 to 400 nucleotides, the mass ratio at which the system displays the highest critical temperature increases from 0 (no RNA) to 0.12 (Fig. 1 *d*). Nevertheless, we observe that the RNA length-dependent effects for polyU–FUS mixtures are significantly smaller than for polyU–PR_{25} mixtures (see Fig. 1, *c* and *d*).

Our results for FUS are consistent with a number of in vitro studies characterizing its RNA concentration-dependent

reentrant phase behavior (41,46). However, that low levels of RNA promote phase separation, while higher RNA levels promote dissolution contrasts with the observation that some condensates are able to form in regions of the cell with very high levels of RNA (up to RNA/protein mass ratios of ~40) (100). Moreover, despite RNA being at high concentrations, it has been shown to play a key role in initiating phase separation of FUS by nucleating protein condensates (20,46,100). For example, RNA plays a prominent role in triggering P-body formation, which occurs as a result of a strong increase in mRNA concentration (100,101). Also, FUS-containing paraspeckles in the nucleus are thought to be nucleated by the long noncoding RNA Neat1, despite the high background RNA concentration (20,46). Even though in vitro studies have not explicitly revealed how the length of RNA defines the maximum RNA concentration that a condensate rich in RNA-binding proteins can take before its stability begins to decrease, there is evidence that indirectly supports this theory: a number of in vitro and in silico studies have identified that minimum RNA lengths are needed to promote phase separation of RNA-binding proteins (43,102) and others have found that shorter RNA molecules are more potent promoters of condensate dissolution (46), in agreement with our results from Fig. 1 *d*. Here, we show directly that increasing the length of the nucleic acid chain increases the capacity of the RNA to promote phase separation and to be incorporated into the condensate at higher RNA concentrations than their shorter counterparts. This result is also supported by in vitro observations of the linker histone H1 having a greater tendency to phase separate with longer RNA molecules compared with shorter ones (103). Therefore, previous findings which have shown that high concentrations of RNA promote condensate dissolution ought to be contextualized with information on the length of the studied nucleic acids. As demonstrated here, it is possible for phase separation to be favored at high RNA concentrations if the RNA chains are long enough to bind multiple proteins simultaneously. Assembling nucleotides in longer RNA chains has the additional advantage of decreasing the electrostatic repulsion among phosphates of separate nucleotides due to the presence of more covalent bonds.

Since our results support the hypothesis that longer RNA molecules are more powerful enhancers of phase separation due to their ability to increase the connectivity of the condensed liquid (66,98), we next investigate this behavior by examining the stability of condensates containing both long and short polyU chains (Fig. 2 *a*). To do so, we simulate mixtures of PR_{25} in the presence of polyU strands of two different lengths, 50 and 400 nt, such that each length represents half of the total polyU mass ratio concentration in each system. For these mixed RNA length systems, we explore polyU/PR_{25} mass ratios ranging from 0.76 to 2.1. We find that mixed RNA length systems display critical

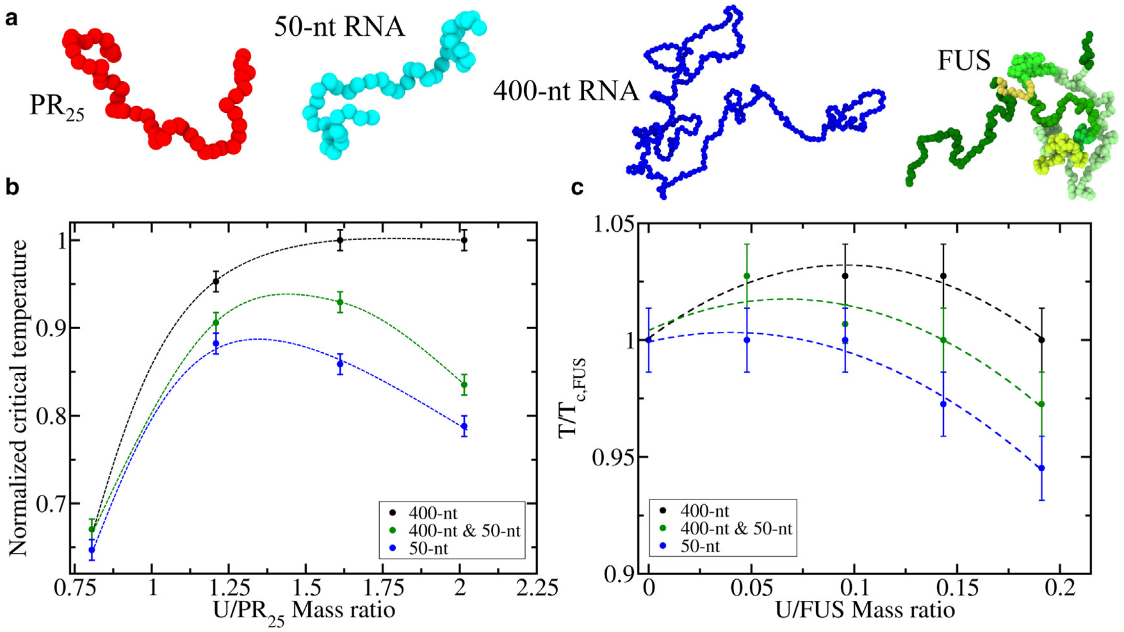

FIGURE 2 RNA-driven reentrant phase behavior of FUS and PR_{25} polyU systems including mixtures with different RNA strand lengths. (*a*) Representation of PR_{25}, FUS, and two RNA strands of 50 and 400 nt each following the same color code and considerations discussed in Fig. 1 *a*. Please note that the size of the beads depicted in this panel has been conveniently rescaled for visualization purposes. (*b*) Normalized critical temperature of polyU-PR_{25} mixtures as a function of the U/PR_{25} mass ratio for different polyU strand lengths as indicated in the legend. (*c*) Normalized critical temperature of FUS-polyU mixtures as a function of the U/FUS mass ratio for different polyU strand lengths as indicated in the legend. For the systems with mixed polyU lengths, each length represents half of the total polyU concentration. While in (*b*) all temperatures have been normalized by the highest T at which phase separation was observed (T = 425 K), in (*c*) all temperatures have been normalized by the critical temperature of pure FUS ($T_{c,FUS}$). To see this figure in color, go online.

temperatures in between those of the pure short and long RNA chain systems, and with the maximum enhancement of critical temperature also occurring at intermediate polyU/PR_{25} mass ratios between that of the pure short and pure long RNA systems (Fig. 2 *b*). We also study FUS-polyU mixtures of 50- and 400-nt polyU strands (such that each length represents half of the total polyU mass ratio concentration) spanning mass ratios from 0 to 0.196. Similar to the results for PR_{25}, the mixed length systems in FUS present intermediate critical temperatures compared with the pure long-chain and short-chain RNA systems, as well as patterns of reentrant phase behavior in which the maximum critical temperature peaks at an intermediate polyU/FUS mass ratio between that of the pure long-chain and short-chain RNA systems (Fig. 2 *c*).

Our findings reveal that mixed length polyU systems are generally more stable than short-chain polyU systems; further emphasizing the ability of long polyU strands to show a stabilizing effect on condensates up to higher concentrations, given that we observe this behavior even when shorter polyU molecules are present. Our observations are consistent with in vitro RNA–FUS phase separation assays reporting that the lncRNA Neat1 (short isoform, length = 3.7 kb) was able to drive reappearance of FUS droplets that had previously been solubilized with tRNA (70–100 nt (104)) (46).

RNA length and concentration determine the internal organization of molecules in RNA–protein condensates and the properties of their interfaces

To rationalize the interaction of RNA length and concentration in modulating the stability of RNA–protein condensates, we now characterize the organization of the different molecules inside the RNA–protein condensates by quantifying the densities of polyU versus proteins across the condensate. These density profiles reveal that there are considerable differences in the distribution of proteins versus polyU depending on the concentration of RNA. Protein-rich systems (i.e., low polyU/PR_{25} mass ratios) form condensates with a surface coated by PR_{25} peptides (Fig. 3, *a* and *b*), whereas the surfaces of polyU-rich droplets (i.e., high polyU/PR_{25} mass ratios) are mostly coated by polyU chains (Fig. 3, *c* and *d*).

In polyU–PR_{25} condensates combining RNAs of different lengths, we find that the RNAs exhibit a striking spatially heterogeneous distribution. The longer 400-nt polyU molecules accumulate in the condensate core, while the shorter 50-nt polyU molecules concentrate preferentially toward the condensate surface (Fig. 3, *a* and *c*). Simulations approximating proteins as patchy colloids have revealed the same heterogeneous organization of high- and low-valency proteins within multicomponent condensates. By looking at the problem from a condensed matter perspective, the simulations revealed that burying high-valency proteins in the center, and exposing low-valency species to the interface, maximizes the enthalpic gain for condensate formation (most bonds of the higher-valency molecules are satisfied at the condensate core) and reduces the interfacial free energy at the droplet interface because the lower-valency molecules act as surfactants (63,66,105). In addition, longer RNAs occupy a smaller excluded volume if they are in the core of the condensate versus if they are localized toward the interface or within the diluted phase. The excluded volume associated with a single 400-nt RNA strand in the condensed phase is expected to be lower than that of eight 50-nt RNA chains. This effect results in a maximization of the system entropy when long RNA strands (compared with short ones) are recruited to the condensate core. Similarly, by preferentially positioning short RNA strands at the interface, the overall RNA excluded volume can be reduced. Hence, both enthalpic and entropic factors likely contribute to explain the heterogeneous arrangement of short and long RNAs within phase-separated condensates shown in Fig. 3, *a* and *c*. In Fig. 3 *e*, we also show that polyU-FUS condensates of mixed RNA length (with equal concentration of 50- and 400-nt strands) display multilayered RNA organization, preferentially locating short polyU chains at the interface and long RNA strands in the core. Such structural organization maximizes at the same time the condensate liquid network connectivity and minimizes the interfacial penalty. We have corroborated that the observed condensate architecture is robust against changes in temperature, which is the variable that globally controls the molecular interaction strength (106) in our simulations. To do so, we computed the same density profiles as in Fig. 3, *a*–*e* at moderately lower and higher temperatures (i.e., 0.85 and 0.95 T/T_c) and found remarkably similar protein and RNA structural arrangements within the condensates (Fig. S1).

Since distinct molecules at the condensate interface can translate into significantly different interfacial properties, such as interfacial free energies (22,43), coalescence fusion rates (107), size-conservation (6,63), or uneven molecule exchange rates (66), we now calculate the interfacial free energy (γ; see supporting material for further details on these calculations). We focus on PR_{25} condensates because converging the value of γ for FUS-based droplets is computationally unfeasible due to the size of FUS (526 amino acids). We start by computing the interfacial free energy for two types of 400-nt polyU–PR_{25} condensates: with a PR_{25}-rich interface (i.e., low U/PR_{25} mass ratio of 0.8) and with a polyU-rich interface (i.e., high U/PR_{25} mass ratio of 1.6), as shown in Fig. 3 *f*. To facilitate the comparison, in both cases we simulate the systems at the same relative temperature ($T/T_c = 0.85$). Strikingly, we find that the condensate with a polyU-rich interface presents a much lower interfacial free energy (almost half) than that with a PR_{25}-rich interface. Such an unexpected finding explains how a high concentration of polyU, beyond the electroneutral point, can boost the stability of condensates: by

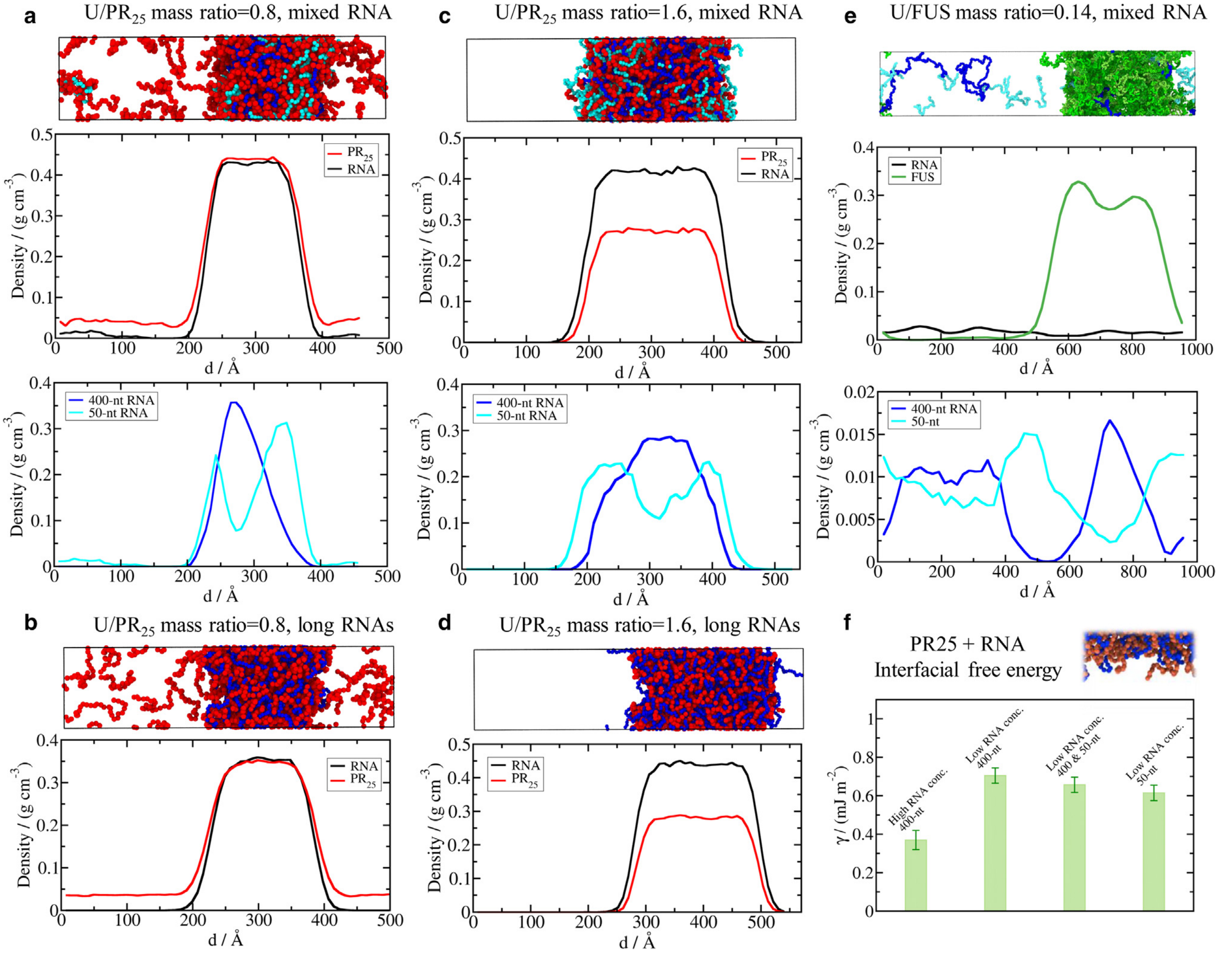

FGURE 3 Structural condensate organization of RNA-binding proteins in the presence of long and short RNA strands. (*a*) PolyU–PR_{25} mixture with a 0.8 U/PR_{25} mass ratio where polyU strands are 50 and 400 nt long (each length contributing half to the total polyU concentration). Top: representative snapshot of a direct coexistence simulation of the system, where PR_{25} molecules are colored in red and long and short RNAs are depicted in blue and cyan, respectively. Middle: density profile of PR_{25} (*red*) and polyU RNA (*black*) along the long axis of the simulation box. Bottom: RNA density profile decomposed in 400-nt (*blue*) and 50-nt (*cyan*) polyU chains. (*b*) The same as in (*a*), but for a polyU–PR_{25} mixture with a U/PR_{25} mass ratio of 0.8 and polyU strands of 400 nt only. Note that we only show one density profile since here all RNAs are of equal length. (*c*) The same as in (*a*), but for a polyU–PR_{25} mixture with a U/PR_{25} mass ratio of 1.6, where polyU strands are also 50 and 400 nt long. (*d*) The same as in (*b*), but for a polyU-PR_{25} mixture with a U/PR_{25} mass ratio of 1.6 and polyU strands of 400 nt only. The temperature of systems shown in (*a–d*) was 0.9 with respect to their corresponding critical temperature. (*e*) RNA–FUS mixture at $T/T_{c,FUS} = 0.98$ and with a U/FUS mass ratio of 0.14, where polyU RNA strands are 50 and 400 nt long (each length contributing half to the total polyU concentration). Top: representative snapshot of a direct coexistence simulation. Middle: density profile of FUS (*green*) and RNA (*black*) along the long axis of the simulation box. Bottom: RNA density profile decomposed in 400-nt (*blue*) and 50-nt (*cyan*) polyU chains. (*f*) Surface tension for different polyU–PR_{25} mixtures, all of them at a temperature of 0.85 with respect to its corresponding critical temperature for the system indicated in the legend. The high RNA concentration corresponds to a U/PR_{25} mass ratio of 1.6, while the low RNA concentration corresponds to a U/PR_{25} mass ratio of 0.8. To see this figure in color, go online.

incorporating a large concentration of RNA, condensates experience a trade-off between the destabilizing effect of a decreased enthalpic gain due to the larger electrostatic repulsion among equally charged nucleotides, and the stabilizing effect of the decrease in the condensate energetic penalty of forming an interface when it is coated with polyU. Our results, therefore, reveal that, in condensates with an excess of polyU, polyU behaves like a surfactant.

To analyze the impact of RNA length, we now calculate the interfacial free energy of two different condensates containing the shorter 50-nt polyU RNA: a PR_{25}, 50- and 400-nt polyU condensate, and a PR_{25} and 50-nt polyU condensate. We estimate the interfacial free energy at the same temperature used above ($T/T_c = 0.85$) and at the lower U/PR_{25} mass ratio of 0.8 ($T/T_c = 0.85$), since imposing a high RNA concentration with short polyU molecules within the condensed phase in a direct coexistence simulation is not feasible. We observe that increasingly adding 50-nt polyU molecules progressively decreases the interfacial free energy of the condensate, with respect to the value of the

400-nt polyU-PR_{25} condensate (Fig. 3 *f*). This observation reinforces the idea that, in RNA-protein condensates that contain RNAs of different lengths, positioning the shorter RNA species at the interface reduces the surface tension as such shorter RNAs act as better surfactants than longer ones (108). Hence, the advantage of mixed length RNA condensates exhibiting multilayered organization results from them presenting similar low surface tensions as those only formed by short RNA strands, while showing considerably higher stability due to long RNA strands increasing the enthalpic gain for condensate formation by contributing more connections to the liquid network connectivity together with PR_{25}.

In addition to the interfacial free energy, we also investigate the molecular contacts that distinct protein domains establish with RNA in RNA–FUS and RNA–PR_{25} droplets. Following the methodology described in (43,71,109), we estimate the frequency of heterotypic polyU–protein interactions at residue-resolution level (Fig. S2). This calculation is performed at $T = 0.95\ T_c$, and in the presence of both long (400-nt) and short (50-nt) RNA strands for both RNA–PR_{25} and RNA–FUS systems (further details of these simulations are provided in the supporting material). While in RNA–PR_{25} droplets RNA–protein interactions are homogeneously distributed across the PR_{25} sequence, in RNA–FUS condensates only specific regions preferentially interact with polyU, such as the RNA-recognition motifs (from the 282nd to the 371st residue), and the three arginine-glycine-rich regions (RGG1: 163 to 267; RGG2: 371 to 422; RGG3: 453 to 526; Fig. S2), in qualitative agreement with experimental observations (34,46,47). These results demonstrate how coarse-grained models, such as the Mpipi, can recapitulate the preferential binding of RNA to certain amino acid motifs within proteins.

Multilayered condensates such as those found in Fig. 3, *a*, *c*, and *e* for polyU–PR_{25} and polyU–FUS mixtures, respectively, can be found across the cell and include FUS-containing paraspeckles (9), stress granules (30), and the nucleolus (6). Indeed, condensate structure and organization has important implications for the behavior of the various components, with those located in the core exhibiting slower exchange rates compared with molecules in the outer shell (66,98). Experiments and simulations reveal that longer RNA strands lead to higher viscosities in RNA–protein condensates (18–20,43,110). Thus, in mixed RNA condensates, a stable core containing long polyU strands is expected to have a higher viscosity, while an outer shell with short polyU strands a lower viscosity (43). Gelification of RNA–protein condensates via fibrillation, with potential pathological implications (21,47,111), has been shown to be seeded at the interface due to a local increase of protein density at the surface (112); hence, incorporating short RNAs into RNA–protein condensates might contribute to preventing their maturation because it decreases the probability of high density fluctuations at the interface.

Self-avoiding polymers trigger concentration-dependent reentrant phase behavior of colloidal patchy-particle condensates modulated by polymer length

To investigate whether the observed impact of RNA length and concentration on the phase behavior of RNA-protein condensates rely on general molecular features such as protein valency, binding affinity, or the relative polymer size/length between proteins and RNA, we employ a minimal coarse-grained model of colloidal patchy particles with self-avoiding polymer chains to mimic proteins and RNA, respectively (89). Iterations of this minimal coarse-grained model have been previously used to investigate critical factors in LLPS such as surface tension, droplet size conservation, or condensate substructure (44,66). The aim of our minimal simulations here is to assess, beyond protein sequence and specific molecular features, the thermodynamic parameters that explain the general differences between the impact of RNA length and concentration on homotypic phase separation versus RNA–protein heterotypic complex coacervation.

We start by computing the phase diagrams of two different types of colloidal patchy particles in the presence of different concentrations and lengths of a self-avoiding polymer that mimics RNA. The first type are patchy particles decorated with three binding sites in a planar arrangement separated by 120° angles (Fig. 4 *a*; see supporting material for further details on the model). Like FUS, these colloidal particles—referred to henceforth as scaffold proteins—are able to phase separate on their own via homotypic interactions below a reduced temperature of $T^* = 0.09$ (89) (see details on reduced units in the supporting material). On the other hand, the second type of patchy colloids possess two binding sites in a polar arrangement, which by construction can only form linear chains and not three-dimensional percolated networks that sustain phase separation (62,64,65) (Fig. 4 *a*). Like PR_{25}, two binding site colloidal particles—referred to henceforth as cognate proteins—cannot phase separate on their own (44). We perform direct coexistence simulations of both scaffold and cognate proteins for different polymer bead/protein ratios using polymer chains of 10, 20, 50, and 100 beads such that the polymer bead/protein ratio is defined in terms of the number of polymer and protein beads, ranging from 0.2 to 1.

First, by focusing on a given polymer bead/protein ratio (i.e., 0.4), we capture the RNA length-dependent enhancement of LLPS reported in our sequence-dependent coarse-grained simulations of PR_{25} and FUS (Fig. 1) where systems containing longer RNA strands can phase separate up to higher critical temperatures (Fig. 4, *b* and *c*). For cognate colloidal particles, increasing the "RNA" chain length from 10 to 100 beads at an RNA/protein ratio of 0.4 leads to a 50% increase in the critical temperature (Fig. 4 *b*). In contrast, the enhancement in critical temperature for the

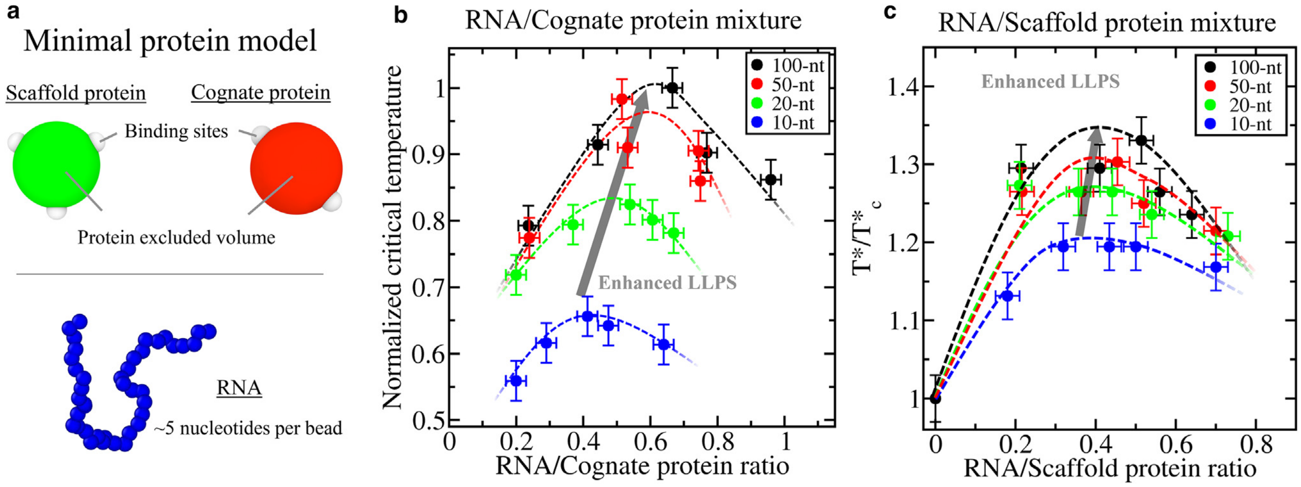

FIGURE 4 Minimal coarse-grained model for protein LLPS. (*a*) Green and red spheres represent the excluded volume of scaffold and cognate proteins, respectively, while gray patches represent the binding sites of the proteins. Two different proteins are modeled: scaffold proteins, with three binding sites in a planar equidistant arrangement, and cognate proteins, with two binding sites in a polar arrangement. Blue spherical beads account for ~5 nt each in the RNA model. Please note that, for visualization purposes, the size of the RNA beads has been scaled down. For further technical details on the model, please see the supporting material. (*b*) Normalized critical temperature of RNA–cognate protein mixtures as a function of the RNA/cognate protein ratio for different RNA strand lengths as indicated in the legend. (*c*) Normalized critical temperature of RNA–scaffold protein mixtures as a function of the RNA/scaffold protein ratio for different RNA strand lengths as indicated in the legend. While in (*b*) all temperatures have been normalized by the highest T ($T^* = 0.11$) at which phase separation was observed, in (*c*) all temperatures have been normalized by the critical temperature of the scaffold protein in the absence of RNA ($T_c^* = 0.09$). To see this figure in color, go online.

same polymer/protein concentration with scaffold proteins is just 8% (Fig. 4 *c*). Clearly, scaffolds show much more subtle dependency on RNA length compared with cognate proteins, in full agreement with our previous sequence-dependent simulations shown in Figs. 1 and 2.

Secondly, by gradually varying the polymer bead/protein ratio (from 0.2 to 1; Fig. 4, *b* and *c*), patterns of concentration-dependent reentrant phase behavior for both types of proteins and distinct RNA polymer lengths can be observed. A moderate increase in the polymer bead/protein ratio leads to an enhancement in the critical temperature, while any further increase in self-avoiding polymer levels results in LLPS inhibition (i.e., lower critical temperatures). For the cognate protein mixtures, we find a significant shift in the ratio that gives the highest critical temperature as a function of polymer length; consistent with our results for PR_{25} with polyU (Fig. 1 *c*). On the other hand, for the scaffold protein mixtures, the shift in the maximum critical temperature toward higher polymer/protein ratios with length is more modest; also in qualitative agreement with our polyU–FUS simulations shown in Fig. 1 *d*. Remarkably, by means of the colloidal patchy particle model, we can explore mixtures with polymer RNA chains much longer than the size of the proteins. We discover that, while condensates with RNA lengths of 50 or 100 beads are stable up to higher critical temperatures compared with those of shorter lengths (i.e., 20 beads), for all polymer bead/protein ratios and for both scaffold and cognate proteins, beyond a certain length at which RNA greatly exceeds the size of the proteins (e.g., 50 times longer than the protein size), the effect of RNA length on LLPS becomes extremely mild (Fig. 4, *b* and *c*); red and black curves). These results also support the notion that the longer RNA strands promote condensate stability by increasing the connectivity among proteins within the condensate network (43,44). Moreover, since even the shortest polymer length added to the scaffold–cognate mixtures meets this criterion, we do not observe the disruptive influence on LLPS of extremely short RNA chains (i.e., 20 nt) with FUS (526 amino acids) observed in Fig. 1 *d*.

Our results are consistent with the experimental observation that longer RNA strands present weaker dissociation constants with the N-RRM1-2 domains of TDP-43 (which, like PR_{25}, cannot phase separate on their own at physiological conditions) than threefold shorter RNA strands (33). Furthermore, it has been shown that length and charge segregation in the IDR domain of VRN1-like proteins has a critical impact on modulating the DNA-induced VRN1 phase separation—where liquid-like, gel-like, or no phase separation behavior can be switched depending on the IDR length and the presence of neutral versus highly charged domains (113). Overall, our patchy particle results presented here highlight that the the impact of RNA length and concentration on the reentrant phase behavior observed for both homotypic and heterotypic phase-separating proteins is a general property of soft-matter systems, such as biomolecules. Therefore, general features of biomolecules, such as their valency, topology, binding affinity, and relative length or size, is what ultimately dictates the intricate phase behavior of multicomponent biomolecular condensates. Such conclusions are supported by our findings using patchy

particle simulations, which highlight the capacity of the minimal model to capture specific RNA-protein condensate behaviors observed with the Mpipi sequence-dependent force field (Fig. 1). For instance, the two approaches consistently predict a much more significant increase in the critical temperature of RNA–PR_{25} condensates with RNA length than that found in RNA–FUS condensates. Such agreement highlights the key role of fundamental physical parameters, such as molecular valency, binding affinity, and polymer length, in the regulation of RNA–protein condensates.

CONCLUSIONS

In this study, using a multiscale modeling approach we reveal how RNA length can tune the concentration-dependent reentrant phase behavior of RNA-protein condensates. Drawing together the results from our sequence-dependent model and patchy particle simulations, we demonstrate that long RNA polymers act as enhancers of phase separation. Not only do longer RNA strands enable phase separation up to higher critical temperatures, they also facilitate phase separation up to higher RNA/protein ratios. We show that this is a physical feature of long polymers, since consistent mass ratios and net charges are used for all our RNA/polymer systems with different lengths. Our finding that longer chain RNA molecules increase the capacity of RNA to raise the critical solution temperature to higher values and that the corresponding condensates can incorporate a higher proportion of RNA nucleotides are consistent with the wide body of work demonstrating the stabilizing role of molecules with an increasing number of repeating chemical building blocks, or higher valencies, on biomolecular condensates (e.g., longer RNAs (44), DNAs (114,16), or polySUMO/polySIM (98), and IDPs with more stickers (35,56,115).

In addition, our finding that long RNA tends to localize to the condensate's core, whereas shorter strands accumulate at the droplet surface, provides further support for the theory that the stabilizing and enabling effects of long RNA chains comes, in part, from their ability to act as nucleators (46) and scaffolds (44) in phase-separated condensates. While long RNA stabilize condensates by increasing the density of molecular connections of the liquid network, short RNAs act as better surfactants that increase condensate stability by reducing their interfacial free energy (63). Moreover, the influence of RNA length on condensate organization and viscoelastic properties has important implications on the dynamics of the different components and the mechanisms explaining liquid-to-solid transitions, with potential implications in neurodegenerative disorders (18,19,21,47,107).

Considering the significant interplay between RNA length and concentration might be relevant to rationalize why some biomolecular condensates, such as stress granules or paraspeckles, can be nucleated by long RNA strands (20,46,100) despite being in RNA-rich environments. According to our results, such behavior might be facilitated by RNAs serving as scaffolds and surfactants of RNA-protein condensates at high concentration. In particular, while high RNA concentrations are expected to inhibit phase separation (53), a diverse population of shorter and longer RNAs can yield stable condensates by incorporating the longer RNAs at concentrations beyond the electroneutral point at the condensate core—to enhance condensate connectivity—and the shorter RNAs at the condensate interface—to reduce the interfacial free energy. The observation that RNA length has a similar impact on the concentration-dependent reentrant phase behavior for both FUS and PR_{25} condensates, although quantitatively shifting the maximum critical temperature by substantially different extents, is also significant and further emphasizes that this observation results from the physical properties of long RNA chains rather than merely on the type of interaction driving phase separation.

Our results also provide thermodynamic and molecular evidence for the hypothesis by Henninger et al. (53) that RNA length and concentration may act in concert to regulate the formation of intranuclear condensates. Specifically, the hypothesis that formation and dissolution of transcriptional condensates is regulated by RNA through a negative feedback loop (53) could be explained by RNA concentration-dependent reentrant phase behavior alone. However, our findings suggesting that longer-chain RNAs, which are produced during a transcriptional burst, stabilize condensates up to higher RNA concentrations may provide robustness to the feedback loop. As higher nucleotide concentrations would need to be reached for condensate dissolution, the transcriptional condensates would remain intact until sufficient levels of longer-chain mRNA have been synthesized.

Taken together, our results provide a thermodynamic view into some of the many ways in which RNA may act as a key regulator of phase separation in the cell and add to the growing consensus considering its role as essential for developing a robust understanding of the regulation and dysregulation of biomolecular condensates (21,116). Several causes of condensate damage in neurodegenerative disorders, such as disease-associated mutations (117,118), do not alter the fundamental changes in phase behavior that differentiate liquid-like condensates from solid-like aggregates (107), but act to increase the likelihood of these changes occurring (111). In other words, the properties of condensates containing mutated proteins might not be discernibly different from those containing wild-type proteins other than in terms of the timescale of their maturation to solid-like aggregates. This suggests that research into potential treatments for condensate-associated diseases may benefit from fundamental biophysical mechanisms of biomolecular condensate regulation (119–122).

SUPPORTING MATERIAL

Supporting material can be found online at https://doi.org/10.1016/j.bpj.2023.03.006.

AUTHOR CONTRIBUTIONS

R.C.-G. and J.R.E. designed the research. I.S.-B. and L.H. performed the research. I.S.-B. and L.H. analyzed the data. I.S.-B., L.H., R.C.-G., and J.R.E. developed new methods. J.R.E. supervised the research. R.C.-G. and J.R.E. wrote the original draft. All authors edited the paper.

ACKNOWLEDGMENTS

This project has received funding from the Oppenheimer Research Fellowship of the University of Cambridge. I.S.-B. acknowledges funding from the Oppenheimer Fellowship, a Derek Brewer scholarship of Emmanuel College, and EPSRC Doctoral Training Program studentship, number EP/T517847/1. J.R.E. also acknowledges funding from the Roger Ekins Research Fellowship of Emmanuel College and the Ramon y Cajal fellowship (RYC2021-030937-I). R.C.-G. acknowledges funding from the European Research Council (ERC) under the European Union Horizon 2020 research and innovation program (grant agreement 803326). This work has been performed using resources provided by the Cambridge Tier-2 system operated by the University of Cambridge Research Computing Service (http://www.hpc.cam.ac.uk) funded by EPSRC Tier-2 capital grant EP/P020259/1.

DECLARATION OF INTERESTS

The authors declare no competing interests.

REFERENCES

1. Hyman, A. A., C. A. Weber, and F. Jülicher. 2014. Liquid-liquid phase separation in biology. *Annu. Rev. Cell Dev. Biol.* 30:39–58.
2. Gomes, E., and J. Shorter. 2019. The molecular language of membraneless organelles. *J. Biol. Chem.* 294:7115–7127.
3. Alberti, S. 2017. Phase separation in biology. *Curr. Biol.* 27:R1097–R1102.
4. Brangwynne, C. P., C. R. Eckmann, …, A. A. Hyman. 2009. Germline P granules are liquid droplets that localize by controlled dissolution/condensation. *Science.* 324:1729–1732.
5. Correll, C. C., J. Bartek, and M. Dundr. 2019. The nucleolus: a multiphase condensate balancing ribosome synthesis and translational capacity in health, aging and ribosomopathies. *Cells.* 8:869.
6. Feric, M., N. Vaidya, …, C. P. Brangwynne. 2016. Coexisting liquid phases underlie nucleolar subcompartments. *Cell.* 165:1686–1697.
7. Platani, M., I. Goldberg, …, A. I. Lamond. 2000. In vivo analysis of cajal body movement, separation, and joining in live human cells. *J. Cell Biol.* 151:1561–1574.
8. Handwerger, K. E., J. A. Cordero, and J. G. Gall. 2005. Cajal bodies, nucleoli, and speckles in the *Xenopus* oocyte nucleus have a low-density, sponge-like structure. *Mol. Biol. Cell.* 16:202–211.
9. West, J. A., M. Mito, …, S. Nakagawa. 2016. Structural, super-resolution microscopy analysis of paraspeckle nuclear body organization. *J. Cell Biol.* 214:817–830.
10. Yamazaki, T., S. Souquere, …, T. Hirose. 2018. Functional domains of NEAT1 architectural lncRNA induce paraspeckle assembly through phase separation. *Mol. Cell.* 70:1038–1053.e7.
11. Bentmann, E., M. Neumann, …, C. Haass. 2012. Requirements for stress granule recruitment of fused in sarcoma (FUS) and TAR DNA-binding protein of 43 kDa (TDP-43). *J. Biol. Chem.* 287:23079–23094.
12. Molliex, A., J. Temirov, …, J. P. Taylor. 2015. Phase separation by low complexity domains promotes stress granule assembly and drives pathological fibrillization. *Cell.* 163:123–133.
13. Strom, A. R., A. V. Emelyanov, …, G. H. Karpen. 2017. Phase separation drives heterochromatin domain formation. *Nature.* 547:241–245.
14. Larson, A. G., D. Elnatan, …, G. J. Narlikar. 2017. Liquid droplet formation by HP1α suggests a role for phase separation in heterochromatin. *Nature.* 547:236–240.
15. Narlikar, G. J. 2020. Phase-separation in chromatin organization. *J. Biosci.* 45:5.
16. Farr, S. E., E. J. Woods, …, R. Collepardo-Guevara. 2021. Nucleosome plasticity is a critical element of chromatin liquid-liquid phase separation and multivalent nucleosome interactions. *Nat Commun.* 12, 2883.
17. Sanulli, S., M. J. Trnka, …, G. J. Narlikar. 2019. HP1 reshapes nucleosome core to promote phase separation of heterochromatin. *Nature.* 575:390–394.
18. Elbaum-Garfinkle, S., Y. Kim, …, C. P. Brangwynne. 2015. The disordered P granule protein LAF-1 drives phase separation into droplets with tunable viscosity and dynamics. *Proc. Natl. Acad. Sci. USA.* 112:7189–7194.
19. Wei, M.-T., S. Elbaum-Garfinkle, …, C. P. Brangwynne. 2017. Phase behaviour of disordered proteins underlying low density and high permeability of liquid organelles. *Nat. Chem.* 9:1118–1125.
20. Burke, K., A. Janke, …, N. Fawzi. 2015. Residue-by-Residue view of In Vitro FUS granules that bind the C-terminal domain of RNA polymerase II. *Mol. Cell.* 60:231–241.
21. Guo, L., and J. Shorter. 2015. It's raining liquids: RNA tunes viscoelasticity and dynamics of membraneless organelles. *Mol. Cell.* 60:189–192.
22. Alshareedah, I., T. Kaur, …, P. R. Banerjee. 2019. Interplay between short-range attraction and long-range repulsion controls reentrant liquid condensation of ribonucleoprotein-RNA complexes. *J. Am. Chem. Soc.* 141:14593–14602.
23. Gui, X., F. Luo, …, B. Dai. 2019. *Nat. Commun.* 10:1.
24. Qamar, S., G. Wang, …, P. St George-Hyslop. 2018. FUS phase separation is modulated by a molecular chaperone and methylation of arginine cation-π interactions. *Cell.* 173:720–734.e15.
25. Murthy, A. C., G. L. Dignon, …, N. L. Fawzi. 2019. Molecular interactions underlying liquid−liquid phase separation of the FUS low-complexity domain. *Nat. Struct. Mol. Biol.* 26:637–648.
26. Rhoads, S., Z. Monahan, …, F. Shewmaker. 2018. The role of post-translational modifications on prion-like aggregation and liquid-phase separation of FUS. *Int. J. Mol. Sci.* 19:886.
27. Yang, P., C. Mathieu, …, J. P. Taylor. 2020. G3BP1 is a tunable switch that triggers phase separation to assemble stress granules. *Cell.* 181:325–345.e28.
28. Gwon, Y., B. A. Maxwell, …, J. P. Taylor. 2021. *Science.* 372:eabf6548.
29. Sanders, D. W., N. Kedersha, …, C. P. Brangwynne. 2020. Competing protein-RNA interaction networks control multiphase intracellular organization. *Cell.* 181:306–324.e28.
30. Guillén-Boixet, J., A. Kopach, …, T. M. Franzmann. 2020. RNA-induced conformational switching and clustering of G3BP drive stress granule assembly by condensation. *Cell.* 181:346–361.e17.
31. Li, H.-R., W.-C. Chiang, …, J.-r. Huang. 2018. TAR DNA-binding protein 43 (TDP-43) liquid–liquid phase separation is mediated by just a few aromatic residues. *J. Biol. Chem.* 293:6090–6098.
32. McGurk, L., E. Gomes, …, N. M. Bonini. 2018. Poly(ADP-Ribose) prevents pathological phase separation of TDP-43 by promoting

liquid demixing and stress granule localization. *Mol. Cell.* 71:703–717.e9.

33. Zacco, E., R. Graña-Montes, …, A. Pastore. 2019. RNA as a key factor in driving or preventing self-assembly of the TAR DNA-binding protein 43. *J. Mol. Biol.* 431:1671–1688.

34. Wang, J., J.-M. Choi, …, A. A. Hyman. 2018. A molecular grammar governing the driving forces for phase separation of prion-like RNA binding proteins. *Cell.* 174:688–699.e16.

35. Bremer, A., M. Farag, …, T. Mittag. 2022. Deciphering how naturally occurring sequence features impact the phase behaviours of disordered prion-like domains. *Nat. Chem.* 14:196–207.

36. Cléry, A., M. Blatter, and F. H. T. Allain. 2008. *Curr. Opin. Struct. Biol.* 18:290–298.

37. Banerjee, P. R., A. N. Milin, …, A. A. Deniz. 2017. Reentrant phase transition drives dynamic substructure formation in ribonucleoprotein droplets. *Angew. Chem. Int. Ed. Engl.* 129:11512–11517.

38. Matsui, S., and R.-S. Nozawa. 2021. RNA impacts formation of biomolecular condensates in the nucleus. *Biomed. Res.* 42:153–160.

39. Kuechler, E. R., P. M. Budzyńska, …, T. Mayor. 2020. Distinct features of stress granule proteins predict localization in membraneless organelles. *J. Mol. Biol.* 432:2349–2368.

40. Chong, P. A., R. M. Vernon, and J. D. Forman-Kay. 2018. RGG/RG motif regions in RNA binding and phase separation. *J. Mol. Biol.* 430:4650–4665.

41. Schwartz, J., X. Wang, …, T. Cech. 2013. RNA seeds higher-order assembly of FUS protein. *Cell Rep.* 5:918–925.

42. Regy, R. M., G. L. Dignon, …, J. Mittal. 2020. Sequence dependent phase separation of protein-polynucleotide mixtures elucidated using molecular simulations. *Nucleic Acids Res.* 48:12593–12603.

43. Tejedor, A. R., A. Garaizar, …, J. R. Espinosa. 2021. 'RNA modulation of transport properties and stability in phase-separated condensates. *Biophys. J.* 120:5169–5186.

44. Sanchez-Burgos, I., J. R. Espinosa, …, R. Collepardo-Guevara. 2022. RNA length has a non-trivial effect in the stability of biomolecular condensates formed by RNA-binding proteins. *PLoS Comput. Biol.* 18:e1009810.

45. Langdon, E. M., Y. Qiu, …, A. S. Gladfelter. 2018. mRNA structure determines specificity of a polyQ-driven phase separation. *Science.* 360:922–927.

46. Maharana, S., J. Wang, …, S. Alberti. 2018. RNA buffers the phase separation behavior of prion-like RNA binding proteins. *Science.* 360:918–921.

47. Portz, B., B. L. Lee, …, J. Shorter. 2021. FUS and TDP-43 phases in health and disease. *Trends Biochem. Sci.* 46:550–563.

48. Vance, C., B. Rogelj, …, C. E. Shaw. 2009. Mutations in FUS, an RNA processing protein, cause familial amyotrophic lateral sclerosis type 6. *Science.* 323:1208–1211.

49. Aumiller, W. M., and C. D. Keating. 2016. Phosphorylation-mediated RNA/peptide complex coacervation as a model for intracellular liquid organelles. *Nat. Chem.* 8:129–137.

50. Fisher, R. S., and S. Elbaum-Garfinkle. 2020. *Nat. Commun.* 11:1.

51. Welsh, T. J., G. Krainer, …, T. P. J. Knowles. 2022. Surface electrostatics govern the emulsion stability of biomolecular condensates. *Nano Lett.* 22:612–621.

52. Lu, T., and E. Spruijt. 2020. Multiphase complex coacervate droplets. *J. Am. Chem. Soc.* 142:2905–2914.

53. Henninger, J. E., O. Oksuz, …, R. A. Young. 2021. RNA-mediated feedback control of transcriptional condensates. *Cell.* 184:207–225.e24.

54. Tunnacliffe, E., and J. R. Chubb. 2020. *Trends Genet.* 36:288–297.

55. Alberti, S., A. Gladfelter, and T. Mittag. 2019. Considerations and challenges in studying liquid-liquid phase separation and biomolecular condensates. *Cell.* 176:419–434.

56. Garaizar, A., I. Sanchez-Burgos, …, J. R. Espinosa. 2020. Expansion of intrinsically disordered proteins increases the range of stability of liquid-liquid phase separation. *Molecules.* 25:4705.

57. Garaizar, A., and J. R. Espinosa. 2021. Salt dependent phase behavior of intrinsically disordered proteins from a coarse-grained model with explicit water and ions. *J. Chem. Phys.* 155:125103.

58. Regy, R. M., J. Thompson, …, J. Mittal. 2021. Improved coarse-grained model for studying sequence dependent phase separation of disordered proteins. *Protein Sci.* 30:1371–1379.

59. Nguemaha, V., and H.-X. Zhou. 2018. Liquid-liquid phase separation of patchy particles illuminates diverse effects of regulatory components on protein droplet formation. *Sci. Rep.* 8:1.

60. Chou, H.-Y., and A. Aksimentiev. 2020. Single-protein collapse determines phase equilibria of a biological condensate. *J. Phys. Chem. Lett.* 11:4923–4929.

61. Ranganathan, S., and E. Shakhnovich. 2021. Effect of RNA on morphology and dynamics of membraneless organelles. *J. Phys. Chem. B.* 125:5035–5044.

62. Bianchi, E., J. Largo, …, F. Sciortino. 2006. Phase diagram of patchy colloids: towards empty liquids. *Phys. Rev. Lett.* 97:168301.

63. Sanchez-Burgos, I., J. A. Joseph, …, J. R. Espinosa. 2021. Size conservation emerges spontaneously in biomolecular condensates formed by scaffolds and surfactant clients. *Sci. Rep.* 11:15241.

64. Espinosa, J. R., A. Garaizar, …, R. Collepardo-Guevara. 2019. Breakdown of the law of rectilinear diameter and related surprises in the liquid-vapor coexistence in systems of patchy particles. *J. Chem. Phys.* 150:224510.

65. Espinosa, J. R., J. A. Joseph, …, R. Collepardo-Guevara. 2020. Liquid network connectivity regulates the stability and composition of biomolecular condensates with many components. *Proc. Natl. Acad. Sci. USA.* 117:13238–13247.

66. Sanchez-Burgos, I., J. R. Espinosa, …, R. Collepardo-Guevara. 2021b. Valency and binding affinity variations can regulate the multilayered organization of protein condensates with many components. *Biomolecules.* 11:278.

67. Statt, A., H. Casademunt, …, A. Z. Panagiotopoulos. 2020. Model for disordered proteins with strongly sequence-dependent liquid phase behavior. *J. Chem. Phys.* 152:075101.

68. Dar, F., and R. Pappu. 2020. *Elife.* 9:e59663.

69. Garaizar, A., J. R. Espinosa, …, R. Collepardo-Guevara. 2022. Aging can transform single-component protein condensates into multiphase architectures. *Proc. Natl. Acad. Sci. USA.* 119:e2119800119.

70. Conicella, A. E., G. L. Dignon, …, N. L. Fawzi. 2020. TDP-43 α-helical structure tunes liquid–liquid phase separation and function. *Proc. Natl. Acad. Sci. USA.* 117:5883–5894.

71. Tejedor, A. R., I. Sanchez-Burgos, …, J. R. Espinosa. 2022. Protein structural transitions critically transform the network connectivity and viscoelasticity of RNA-binding protein condensates but RNA can prevent it. *Nat. Commun.* 13:5717.

72. Jacobs, W. M. 2021. Self-Assembly of biomolecular condensates with shared components. *Phys. Rev. Lett.* 126:258101.

73. Joseph, J. A., A. Reinhardt, …, R. Collepardo-Guevara. 2021. Physics-driven coarse-grained model for biomolecular phase separation with near-quantitative accuracy. *Nat. Comput. Sci.* 1:732–743.

74. Krainer, G., T. J. Welsh, …, T. P. J. Knowles. 2021. Reentrant liquid condensate phase of proteins is stabilized by hydrophobic and non-ionic interactions. *Nat. Commun.* 12:1085.

75. Lee, J., Y. O. Popov, and G. H. Fredrickson. 2008. Complex coacervation: a field theoretic simulation study of polyelectrolyte complexation. *J. Chem. Phys.* 128:224908.

76. Lin, Y.-H., J. P. Brady, …, H. S. Chan. 2017. Charge pattern matching as a 'fuzzy' mode of molecular recognition for the functional phase separations of intrinsically disordered proteins. *New J. Phys.* 19:115003.

77. McCarty, J., K. T. Delaney, …, J.-E. Shea. 2019. Complete phase diagram for liquid-liquid phase separation of intrinsically disordered proteins. *J. Phys. Chem. Lett.* 10:1644–1652.
78. Choi, J.-M., A. A. Hyman, and R. V. Pappu. 2020. Generalized models for bond percolation transitions of associative polymers. *Phys. Rev. E.* 102:042403.
79. Weber, C. A., D. Zwicker, …, C. F. Lee. 2019. Physics of active emulsions. *Rep. Prog. Phys.* 82:064601.
80. Harmon, T. S., A. S. Holehouse, …, R. V. Pappu. 2017. *Elife.* 6:e30294.
81. Das, S., A. Eisen, …, H. S. Chan. 2018. A lattice model of charge-pattern-dependent polyampholyte phase separation. *J. Phys. Chem. B.* 122:5418–5431.
82. Harmon, T. S., A. S. Holehouse, and R. V. Pappu. 2018. Differential solvation of intrinsically disordered linkers drives the formation of spatially organized droplets in ternary systems of linear multivalent proteins. *New J. Phys.* 20:045002.
83. Choi, J.-M., F. Dar, and R. V. Pappu. 2019. LASSI: a lattice model for simulating phase transitions of multivalent proteins. *PLoS Comput. Biol.* 15:e1007028.
84. Dignon, G. L., W. Zheng, …, J. Mittal. 2018. Relation between single-molecule properties and phase behavior of intrinsically disordered proteins. *Proc. Natl. Acad. Sci. USA.* 115:9929–9934.
85. Dignon, G. L., W. Zheng, …, J. Mittal. 2018. Sequence determinants of protein phase behavior from a coarse-grained model. *PLoS Comput. Biol.* 14:e1005941.
86. Perdikari, T. M., N. Jovic, …, J. Mittal. 2021. A predictive coarse-grained model for position-specific effects of post-translational modifications. *Biophys. J.* 120:1187–1197. https://doi.org/10.1016/j.bpj.2021.01.034.
87. Tesei, G., T. K. Schulze, …, K. Lindorff-Larsen. 2021. *Proc. Natl. Acad. Sci. USA.* 118:e2111696118.
88. Qi, Y., and B. Zhang. 2021. *Nat. Commun.* 12:1.
89. Joseph, J. A., J. R. Espinosa, …, R. Collepardo-Guevara. 2021. Thermodynamics and kinetics of phase separation of protein-RNA mixtures by a minimal model. *Biophys. J.* 120:1219–1230.
90. Wang, X., S. Ramírez-Hinestrosa, …, D. Frenkel. 2020. The Lennard-Jones potential: when (not) to use it. *Phys. Chem. Chem. Phys.* 22:10624–10633.
91. Ahlers, J., E. M. Adams, …, M. Havenith. 2021. The key role of solvent in condensation: mapping water in liquid-liquid phase-separated FUS. *Biophys. J.* 120:1266–1275.
92. Regy, R. M., G. L. Dignon, …, J. Mittal. 2020. *bioRxiv.*
93. Jover, J., A. J. Haslam, …, E. A. Müller. 2012. Pseudo hard-sphere potential for use in continuous molecular-dynamics simulation of spherical and chain molecules. *J. Chem. Phys.* 137:144505. https://doi.org/10.1063/1.4754275.
94. Espinosa, J. R., C. Vega, and E. Sanz. 2014. The mold integration method for the calculation of the crystal-fluid interfacial free energy from simulations. *J. Chem. Phys.* 141:134709.
95. Ladd, A., and L. Woodcock. 1977. Triple-point coexistence properties of the Lennard-Jones system. *Chem. Phys. Lett.* 51:155–159.
96. García Fernández, R., J. L. F. Abascal, and C. Vega. 2006. The melting point of ice Ih for common water models calculated from direct coexistence of the solid-liquid interface. *J. Chem. Phys.* 124:144506.
97. Rowlinson, J. S., and B. Widom. 2013. Molecular Theory of Capillarity. Courier Corporation.
98. Banani, S. F., A. M. Rice, …, M. K. Rosen. 2016. Compositional control of phase-separated cellular bodies. *Cell.* 166:651–663.
99. Kang, J., L. Lim, and J. Song. 2019. ATP binds and inhibits the neurodegeneration-associated fibrillization of the FUS RRM domain. *Commun. Biol.* 2:223.
100. Van Treeck, B., and R. Parker. 2018. Emerging roles for intermolecular RNA-RNA interactions in RNP assemblies. *Cell.* 174:791–802.
101. Teixeira, D., U. Sheth, …, R. Parker. 2005. Processing bodies require RNA for assembly and contain nontranslating mRNAs. *RNA.* 11:371–382.
102. Lee, Y.-B., H.-J. Chen, …, C. Shaw. 2013. Hexanucleotide repeats in ALS/FTD form length-dependent RNA foci, sequester RNA binding proteins, and are neurotoxic. *Cell Rep.* 5:1178–1186.
103. Leicher, R., A. Osunsade, …, S. Liu. 2022. Single-stranded nucleic acid binding and coacervation by linker histone H1. *Nat. Struct. Mol. Biol.* 29:463–471.
104. Krahn, N., J. T. Fischer, and D. Söll. 2020. Naturally occurring tRNAs with non-canonical structures. *Front. Microbiol.* 2616.
105. Palaia, I., and A. Šarić. 2022. Controlling cluster size in 2D phase-separating binary mixtures with specific interactions. *J. Chem. Phys.* 156:194902.
106. Lappala, A., L. Sefton, …, E. M. Terentjev. 2019. Connectivity and free-surface effects in polymer glasses. *Sci. Rep.* 9:3830.
107. Garaizar, A., J. R. Espinosa, …, R. Collepardo-Guevara. 2022. Kinetic interplay between droplet maturation and coalescence modulates shape of aged protein condensates. *Sci. Rep.* 12:4390.
108. Cochard, A., M. Garcia-Jove Navarro, …, Z. Gueroui. 2022. RNA at the surface of phase-separated condensates impacts their size and number. *Biophys. J.* 121:1675–1690.
109. Blazquez, S., I. Sanchez-Burgos, …, J. R. Espinosa. 2022. Location and concentration of aromatic-rich segments dictates the percolating inter-molecular network and viscoelastic properties of ageing condensates. *Preprint at bioRxiv.*
110. Zhang, H., S. Elbaum-Garfinkle, …, A. Gladfelter. 2015. RNA controls PolyQ protein phase transitions. *Mol. Cell.* 60:220–230.
111. Patel, A., H. O. Lee, …, S. Alberti. 2015. A liquid-to-solid phase transition of the ALS protein FUS accelerated by disease mutation. *Cell.* 162:1066–1077.
112. Shen, Y., A. Chen, …, T. P. J. Knowles. 2022. Solid/liquid coexistence during aging of FUS condensates. Preprint at bioRxiv. https://doi.org/10.1101/2022.08.15.503964.
113. Wang, Y., H. Zhou, …, L. Lai. 2021. Charge segregation in the intrinsically disordered region governs VRN1 and DNA liquid-like phase separation robustness. *J. Mol. Biol.* 433:167269.
114. Muzzopappa, F., M. Hertzog, and F. Erdel. 2021. DNA length tunes the fluidity of DNA-based condensates. *Biophys. J.* 120:1288–1300.
115. Martin, E. W., A. S. Holehouse, …, T. Mittag. 2020. Valence and patterning of aromatic residues determine the phase behavior of prion-like domains. *Science.* 367:694–699.
116. Zhu, H., H. Fu, …, X. Shen. 2022. RNAPhaSep: a resource of RNAs undergoing phase separation. *Nucleic Acids Res.* 50:D340–D346.
117. Boeynaems, S., E. Bogaert, …, L. Van Den Bosch. 2017. Phase separation of C9orf72 dipeptide repeats perturbs stress granule dynamics. *Mol. Cell.* 65:1044–1055.e5.
118. Renton, A. E., A. Chiò, and B. J. Traynor. 2014. State of play in amyotrophic lateral sclerosis genetics. *Nat. Neurosci.* 17:17–23.
119. Wang, B., L. Zhang, …, F. Zhou. 2021. *Signal Transduct. Targeted Ther.* 6:1.
120. Alberti, S., and D. Dormann. 2019. Liquid–liquid phase separation in disease. *Annu. Rev. Genet.* 53:171–194.
121. Wheeler, R. J. 2020. Therapeutics—how to treat phase separation-associated diseases. *Emerg. Top. Life Sci.* 4:331–342.
122. Verdile, V., E. De Paola, and M. P. Paronetto. 2019. *Front. Genet.* 10:173.

Biophysical Journal, Volume *122*

Supplemental information

Surfactants or scaffolds? RNAs of varying lengths control the thermodynamic stability of condensates differently

Ignacio Sanchez-Burgos, Lara Herriott, Rosana Collepardo-Guevara, and Jorge R. Espinosa

Supplementary Material: Surfactants or scaffolds? RNAs of different lengths exhibit heterogeneous distributions and play diverse roles in RNA-protein condensates

Ignacio Sanchez-Burgos[‡,1], Lara Herriott[‡,1], Rosana Collepardo-Guevara[2,3,*] and Jorge R. Espinosa[1,4,*]

[1] Maxwell Centre, Cavendish Laboratory, Department of Physics, University of Cambridge, J J Thomson Avenue, Cambridge CB3 0HE, United Kingdom.

[2] Yusuf Hamied Department of Chemistry, University of Cambridge, Lensfield Road, Cambridge CB2 1EW, United Kingdom

[3] Department of Genetics, University of Cambridge, Cambridge, CB2 3EH

[4] Departament of Chemical Physics, Faculty of Chemical Sciences, Universidad Complutense de Madrid, 28040 Madrid, Spain.

‡ = *These authors contributed equally to this work,* * = *To whom correspondence should be sent.*

(Dated: 2nd March 2023)

SI. MODELS AND SIMULATION DETAILS

A. Mpipi Model

For our simulations of *fused in sarcoma* (FUS) (see sequence below) and PR_{25} with polyU RNA, we employ the high-resolution sequence-dependent coarse-grained model Mpipi [1], which describes almost quantitatively the temperature-dependent LLPS phase behaviour of different protein condensates such as that of FUS. In addition, this model correctly predicts the multiphase behaviour of the polyR/polyK/polyU system, and recapitulates experimental LLPS trends for sequence mutations on FUS, DDX4 NTD and LAF-1 RRG domain variants [1]. Within this force field, electrostatic interactions are modelled through a Coulombic term with Debye–Huckel electrostatic screening [2], given by the sum over all particle-particle (i, j) interactions as:

$$E_{elec} = \sum_{i,j} \frac{q_i q_j}{4\pi\epsilon_r\epsilon_0 r_{ij}} exp(-\kappa r_{ij}) \tag{S1}$$

where q is the charge (being -0.75e for the different nucleotides: A, C, G, U; and +0.75e for amino acids such as R and K, +0.375e for H, and -0.75e for D and E residues), $\epsilon_r = 80$ is the relative dielectric constant of water, ϵ_0 is the electric constant, $\kappa^{-1} = 795$ pm is the Debye screening length, and r_{ij} is the distance separating particles i and j. For these interactions, a Coulomb cut-off of 3.5 nm is employed. The rest of non-bonded interactions (i.e., hydrophobic, cation-π or π-π) between distinct protein/RNA beads are modelled via the Wang–Frenkel potential [3]:

$$E_{WF} = \sum_{i,j} \epsilon_{ij}\alpha_{ij} \left[\left(\frac{\sigma_{ij}}{r_{ij}}\right)^{2\mu_{ij}} - 1\right] \left[\left(\frac{\sigma_{ij}}{r_{ij}}\right)^{2\mu_{ij}} - 1\right]^{2\nu_{ij}} \tag{S2}$$

where

$$\alpha_{ij} = 2\nu_{ij} \left(\frac{R_{ij}}{\sigma_{ij}}\right)^{2\mu_{ij}} \left[\frac{2\nu_{ij}+1}{2\nu_{ij}\left[\left(\frac{R_{ij}}{\sigma_{ij}}\right)^{2\mu_{ij}} - 1\right]}\right]^{2\nu_{ij}+1} \tag{S3}$$

representing σ_{ij} the molecular diameter of each residue/nucleotide and ϵ_{ij} the interaction strength between distinct amino acids and nucleotides (i and j). While σ_{ij}, ϵ_{ij} and μ_{ij} are parameters specified for each pair of interactions (see Ref. [1]), ν_{ij} and R_{ij} are constant model parameters set to $\nu_{ij} = 1$ and $R_{ij} = 3\sigma_{ij}$ for every interaction. Finally, bond energy is computed with an harmonic bond potential of the following form:

$$E_{bond} = \sum_b \frac{1}{2} k(r_b - r_0) \tag{S4}$$

where b is the total number of bonds, r_b is the bond distance, k=8.03 $\mathrm{Jmol^{-1}pm^{-2}}$ is the spring constant and r_0 is the bond reference position, set to 381 pm and 500 pm for protein and RNA bonds respectively. For further details on this force field and the full list of the model parameters please see Ref. [1].

B. FUS Sequence and PDB of the structured domains

Full-FUS sequence

MASNDYTQQATQSYGAYPTQPGQGYSQQSSQPYGQQSYSGYSQSTDTSGYGQSSYSSYGQSQNTGYGTQSTPQGYGSTGGYGSS
QSSQSSYGQQSSYPGYGQQPAPSSTSGSYGSSSQSSSYGQPQSGSYSQQPSYGGQQQSYGQQQSYNPPQGYGQQNQYNSSSGGGG
GGGGGGNYGQDQSSMSSGGGSGGGYGNQDQSGGGGSGGYGQQDRGGRGRGGSGGGGGGGGGGYNRSSGGYEPRGRGGGRG
GRGGMGGSDRGGFNKFGGPRDQGSRHDSEQDNSDNNTIFVQGLGENVTIESVADYFKQIGIIKTNKKTGQPMINLYTDRETGKL
KGEATVSFDDPPSAKAAIDWFDGKEFSGNPIKVSFATRRADFNRGGGNGRGGRGRGGPMGRGGYGGGGSGGGGRGGFPSGGG
GGGGQQRAGDWKCPNPTCENMNFSWRNECNQCKAPKPDGPGGGPGGSHMGGNYGDDRRGGRGGYDRGGYRGRGGDRGGF
RGGRGGGDRGGFGPGKMDSRGEHRQDRRERPY

The following Protein Data Bank (PDB) codes were used to build the globular structured domains of FUS (residues from 285–371 (PDB code: 2LCW) and from 422–453 (PDB code: 6G99)).

C. Colloidal patchy particle model for protein/RNA phase-separation

For the minimal coarse-grained simulations shown in Fig. 4 of the main text, we employ a patchy particle model [4–7] in which proteins are described by a pseudo hard-sphere (PHS) potential [8] that accounts for their excluded volume:

$$E_{PHS} = \sum_{i<j} \begin{cases} \lambda_r (\frac{\lambda_r}{\lambda_a})^{\lambda_a} \varepsilon_R \left[(\frac{\sigma}{r_{ij}})^{\lambda_r} - (\frac{\sigma}{r_{ij}})^{\lambda_a} \right] + \varepsilon_R; & \text{if } r < (\frac{\lambda_r}{\lambda_a})\sigma \\ 0; & \text{if } r \geq (\frac{\lambda_r}{\lambda_a})\sigma \end{cases}. \tag{S5}$$

where λ_a =49 and λ_r=50 are the exponents of the attractive and repulsive terms respectively, and ε_R accounts for the energy shift of the pseudo hard-sphere interaction. On top of this, we add a continuous square-well (CSW) potential for modeling the different protein binding sites, therefore mimicking protein multivalency:

$$E_{CSW} = \sum_{i<j} -\frac{1}{2}\epsilon_{CSW} \left[1 - tanh(\frac{r_{ij} - r_w}{\alpha}) \right] \tag{S6}$$

where ϵ_{CSW} is the depth of the potential energy well, r_w the radius of the attractive well, and α controls the steepness of the well. We choose $\alpha = 0.005\sigma$ and $r_w = 0.12\sigma$ so that each binding site can only interact with another single one. RNA-protein interactions are modeled with a standard Lennard-Jones (LJ) potential [9]:

$$E_{LJ} = \sum_{i<j} 4\epsilon_{LJ} \left[\left(\frac{\sigma}{r_{ij}} \right)^{12} - \left(\frac{\sigma}{r_{ij}} \right)^{6} \right] \tag{S7}$$

where ϵ_{LJ} measures the depth of potential and σ the excluded volume between proteins and RNA. The Lennard-Jones potential is employed between protein cores (not binding sites) and RNA beads, while RNA-RNA interactions are modelled via the PHS potential [8], with a repulsive Yukawa potential on top of the PHS one:

$$E_{Yukawa} = \sum_{i<j} A \frac{e^{-\kappa r_{ij}}}{r_{ij}} \tag{S8}$$

where we set A at 0.42 kcal·Å / mol and κ at 2.57 Å^{-1}. In this way, we model RNA as a self-repulsive polymer of bonded repulsive spheres, and protein-RNA interactions via sites that are not the protein-protein binding sites. Hence, if one protein is bonded to RNA, it can still bind to other proteins. In our model, the mass of each patch is 5% of the central PHS particle mass, which is set to 3.32x10^{-26}kg, despite this choice being irrelevant for equilibrium simulations. This 5% ratio fixes the moment of inertia of the patchy particles (our minimal coarse-grained proteins). The molecular diameter of the proteins, both scaffold and cognate proteins, as well as the RNA beads is $\sigma = 0.3405$

nm, and the value of ε_R/k_B is 119.81K. With this model, we express magnitudes in reduced units: reduced temperature is defined as $T^* = k_BT/\epsilon_{CSW}$, reduced density as $\rho^* = (N/V)\sigma^3$, reduced pressure as $p^* = p\sigma^3/(k_BT)$, and reduced time as $\sqrt{\sigma^2 m/(k_BT)}$. In order to keep the PHS interaction as similar as possible to a pure HS interaction, we fix k_BT/ε_R at a value of 1.5 as suggested in Ref. [8] (fixing $T = 179.71$K). We then control the effective strength of the binding protein attraction by varying ϵ_{CSW} such that the reduced temperature, $T^* = k_BT/\epsilon_{CSW}$, is of the order of $\mathcal{O}(0.1)$. The cut-off distance for the interactions in this model are 1.17σ for both PHS and CSW potentials and 5σ for the LJ interactions. The ϵ_{LJ}/k_B for LJ interactions is set to 152.5K.

This model has been proven to qualitatively reproduce the effect of protein valency in LLPS [5], the enhancement of RNA-mediated LLPS in RNA-binding proteins [10] or the multilayer organization [6] and condensate size conservation in scaffold-client mixtures [7].

D. Simulation details

Our Direct Coexistence simulations [11, 12] are performed in the NVT ensemble (i.e. constant number of particles (N), volume (V) and temperature (T)), for which we use a Nosé–Hoover thermostat [13, 14] with a relaxation time of 5 ps for the Mpipi model simulations and 0.074 in reduced units for the patchy particle simulations. Since all our potentials are continuous and differentiable, we perform all our simulations using the LAMMPS Molecular Dynamics package [15]. Periodic boundary conditions are used in the three directions of space. The timestep chosen for the Verlet integration of the equations of motion is 10 fs for the Mpipi model and 3.7x10^{-4} in reduced time units for the patchy particle model. For the bulk simulations with the Mpipi model, we employ the NpT ensemble, controlling the pressure (p) with the Nosé–Hoover barostat [16] using a relaxation time of 10 ps. All our simulations were equilibrated for at least 50 ns, ensuring that quantities such as the potential energy of the system and the condensate density remain constant over time. Production runs generally last from 200 ns up to few microseconds depending on the system and the property of interest, being more computationally demanding those simulations aiming to elucidate the condensate architecture (as shown in Figs. 3 (main text) and S1).

PR_{25}-polyU simulations were performed with 96 repeats of the protein and varying amounts of RNA, ranging from 1600 to 4800 nucleotides in total, and split into different chains to achieve the desired mass ratio and polyU strand length. For FUS-polyU simulations, we make use of 48 protein replicas and 400 to 1600 nucleotides in total forming RNA chains of different lengths. In the minimal model simulations we made use of 1000 coarse-grained proteins along with 100 to 600 RNA beads in total split into chains of different lengths as indicated in the main text.

SII. COMPUTING PHASE DIAGRAMS VIA DIRECT COEXISTENCE

To calculate the coexisting densities of the phase diagrams shown in Fig. 1b and 4b-c of the main text, we employ the Direct Coexistence method [11, 12, 17]. Within this scheme, the two coexisting phases are simulated by preparing periodically extended slabs of the two phases, the condensed and the diluted one, in the same simulation box. We use an implicit solvent model; accordingly, the diluted phase (protein-poor liquid phase) and the condensed phase (protein-rich liquid phase) are effectively a vapour and a liquid phase, respectively. Once our DC simulations have reached equilibrium, we compute the density profile along the long axis of the box, and thus, we extract the density of the two coexisting phases (as shown in Fig. 1(b) and 3(a-e) of the main text and the Supporting Material of Ref. [10]). From the plateau of the condensed phase and the diluted one, we measure the density (avoiding the interfaces between both phases). To estimate the critical point of the phase diagrams, we use the universal scaling law of coexistence densities near a critical point [18], and the law of rectilinear diameters [19]:

$$\big(\rho_l(T) - \rho_v(T)\big)^{3.06} = d\left(1 - \frac{T}{T_c}\right) \tag{S9}$$

and

$$(\rho_l(T) + \rho_v(T))/2 = \rho_c + s_2(T_c - T) \tag{S10}$$

where ρ_l and ρ_v refer to the coexisting densities of the condensed and diluted phases respectively, while ρ_c is the critical density, T_c is the critical temperature, and d and s_2 are fitting parameters. On the other hand, the methodology to estimate critical temperatures via NpT bulk simulations is provided in Section II of the main text, and illustrated in Fig. 1b.

SIII. COMPUTING THE INTERFACIAL FREE ENERGY FROM DIRECT COEXISTENCE SIMULATIONS

From Direct Coexistence simulations, we can obtain the interfacial free energy (γ) of the condensates by using the following expression:

$$\gamma = \int_{-\infty}^{\infty} [p_N(x) - p_T(x)]\, dx = \frac{L_x}{2N}(\overline{p}_N - \overline{p}_T) \tag{S11}$$

where L_x corresponds to the long side of the simulation box (perpendicular to the slab interfaces), N is the number of droplets in the system, and p_N and p_T are the normal and tangential components of the pressure tensor with respect to the interfaces of the system (note that the tangential component must be averaged over the two tangential directions).

SIV. SIMULATION CONVERGENCE

To ensure that our simulations reach convergence, we estimate from our bulk NpT simulations (presented in Fig. 1 and 2 of the main text) the approximate relaxation timescale for the systems to equilibrate. This can be easily approximated from the time that it takes the centre-of-mass particle of the largest biomolecule in the system to diffuse at least 1 or 2 times its average (protein or RNA) radius of gyration (as discussed in Refs. [20–22]). Once simulated the timescale needed to diffuse such distance (typically between 3 to 5 microseconds), we compute density profiles of the system over time, and we average the profiles from independent blocks to determine the equilibrium condensate structural arrangement (as shown in Fig. 3). Importantly, we note that the impact of a modest equilibration in systems employed to determine the critical temperature can be less dramatic than that required for characterizing the condensate architecture as shown in Fig. 3 of the main text, and Fig S1.

SV. EFFECT OF TEMPERATURE ON THE RNA-PROTEIN CONDENSATE STRUCTURAL ARRANGEMENT

In Fig. 3 of the main text, we present density profiles for FUS-RNA and PR_{25}-RNA condensates at T=0.98T_c. In order to assess the effect of temperature on the observed condensate architecture, in Fig. S1 we show density profiles for the same condensates but at T/T_c 0.95 and 0.85 to demonstrate the mild dependence of condensate organization on temperature. We observe that there is no significant variation with temperature in the local arrangement of proteins and RNA along the condensate, with the density profiles presented in Fig. S1 being very similar to those of Fig. 3 in the main text.

SVI. PROTEIN-RNA CONTACT FREQUENCY MAPS

As mentioned in the main text, the Mpipi model, despite being a residue-resolution coarse-grained model, it is able to qualitatively recapitulate the different contact frequencies that distinct protein domains can establish with RNA [1]. Here, following the methodology described in Refs. [21–23], we evaluate the contact frequency between polyU RNA strands and the different protein amino acids of both FUS and PR_{25} within the condensates. In Figure S2, we show the contact map frequency of the different residues in both FUS-RNA and PR_{25}-RNA condensates for the heterotypic protein-RNA interactions. This calculation has been performed at T=0.95T_c and in the presence of both long (400-nt) and short (50-nt) RNA chains for both PR_{25}-RNA and FUS-RNA systems (i.e., same conditions of the results shown in Fig. S1 for T=0.95T_c). For the case of the PR_{25}-RNA system, the U/PR_{25} mass ratio was 1.6, while for the FUS-RNA condensate it was U/FUS mass ratio = 0.14. While in PR_{25}-RNA droplets protein-RNA

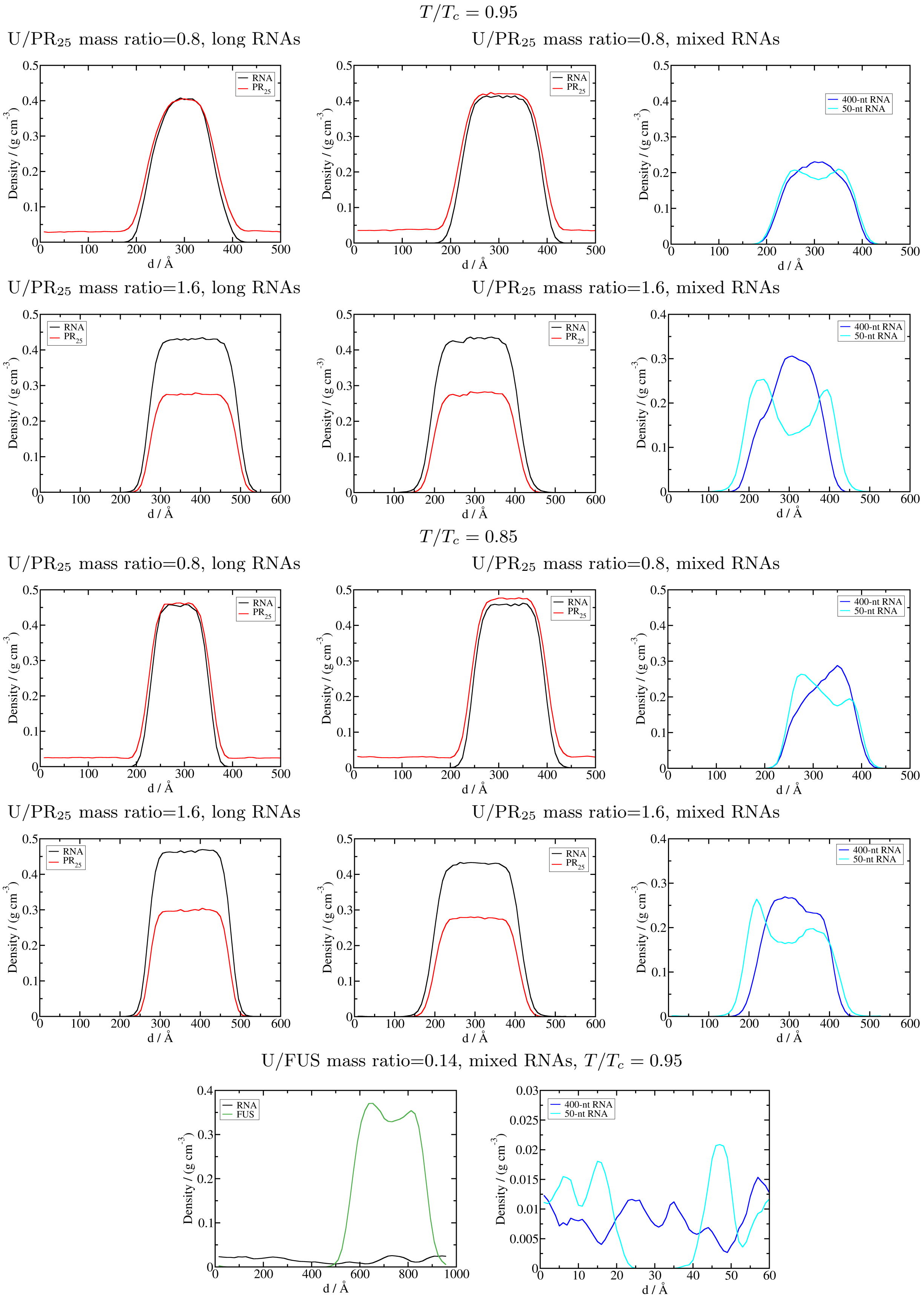

Figure S1: RNA-protein density profiles for the condensates shown in Fig. 3 of the main text and at the temperatures indicated in the different panels.

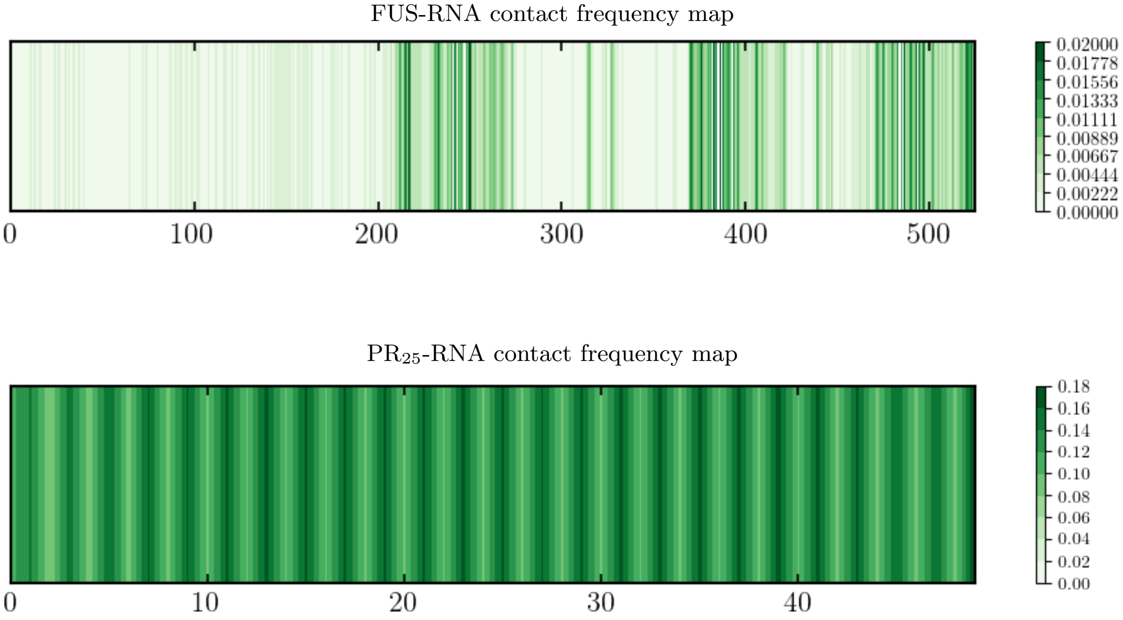

Figure S2: Contact probability per residue between polyU and the different amino acids composing FUS-RNA (Top panel) and PR_{25}-RNA (Bottom panel) condensates. The bar indicates the frequency of amino acid-U contacts (normalized by the averaged total number of protein-RNA contacts), with the darkest colours corresponding to a higher number of contacts. Calculations were performed at $T=0.95T_c$ (T_c being the corresponding critical temperature of each system) and in the presence of both long (400-nt) and short (50-nt) RNA chains for both PR_{25}-RNA and FUS-RNA systems (as studied in Fig. S1). For the case of the PR_{25}-RNA system, we select a U/PR_{25} mass ratio of 1.6, while for the FUS-RNA a U/FUS mass ratio of 0.14.

interactions are quite homogeneous due to the dipeptide repeat distribution of the PR_{25} sequence, in FUS-RNA condensates, the domains which preferentially interact with polyU are the RNA-recognition motifs (from the 282nd to the 371st residue), and the three arginine-glycine rich regions (RGG1: 163 to 267; RGG2: 371 to 422; RGG3: 453 to 526), in qualitative agreement with experimental observations [24–26].

---

[1] A. J. Joseph, A. Reinhardt, A. Aguirre, P. Y. Chew, K. O. Russell, J. R. Espinosa, A. Garaizar, and R. Collepardo-Guevara, Nat. Comput. Sci. , in press (2021).

[2] P. Debye and E. Hückel, Physikalische Zeitschrift **24**, 185 (1923).

[3] X. Wang, S. Ramírez-Hinestrosa, J. Dobnikar, and D. Frenkel, Physical Chemistry Chemical Physics **22**, 10624 (2020).

[4] J. R. Espinosa, A. Garaizar, C. Vega, D. Frenkel, and R. Collepardo-Guevara, J. Chem. Phys **150**, 224510 (2019).

[5] J. R. Espinosa, J. A. Joseph, I. Sanchez-Burgos, A. Garaizar, D. Frenkel, and R. Collepardo-Guevara, Proceedings of the National Academy of Sciences (2020).

[6] I. Sanchez-Burgos, J. R. Espinosa, J. A. Joseph, and R. Collepardo-Guevara, Biomolecules **11**, 278 (2021).

[7] I. Sanchez-Burgos, J. A. Joseph, R. Collepardo-Guevara, and J. R. Espinosa, bioRxiv (2021).

[8] J. Jover, A. J. Haslam, A. Galindo, G. Jackson, and E. A. Müller, Journal of Chemical Physics **137** (2012), 10.1063/1.4754275.

[9] J. E. Jones, Proceedings of the Royal Society of London. Series A, Containing Papers of a Mathematical and Physical Character **106**, 441 (1924).

[10] J. A. Joseph, J. R. Espinosa, I. Sanchez-Burgos, A. Garaizar, D. Frenkel, and R. Collepardo-Guevara, Biophysical Journal **120**, 1219 (2021).

[11] A. J. Ladd and L. V. Woodcock, Chemical Physics Letters **51**, 155 (1977).

[12] R. García Fernández, J. L. F. Abascal, and C. Vega, The Journal of Chemical Physics **124**, 144506 (2006).

[13] S. Nosé, The Journal of Chemical Physics **81**, 511 (1984).

[14] W. G. Hoover, Phys. Rev. A **31**, 1695 (1985).
[15] S. Plimpton, Journal of Computational Physics **117**, 1 (1995).
[16] W. G. Hoover, Physical Review A **34**, 2499 (1986).
[17] J. R. Espinosa, E. Sanz, C. Valeriani, and C. Vega, Journal of Chemical Physics **139** (2013), 10.1063/1.4823499.
[18] J. S. Rowlinson and B. Widom, *Molecular theory of capillarity* (Courier Corporation, 2013).
[19] J. A. Zollweg and G. W. Mulholland, The Journal of Chemical Physics **57**, 1021 (1972).
[20] A. Garaizar, J. R. Espinosa, J. A. Joseph, and R. Collepardo-Guevara, Scientific reports **12**, 4390 (2022).
[21] A. R. Tejedor, I. Sanchez-Burgos, M. Estevez-Espinosa, A. Garaizar, R. Collepardo-Guevara, J. Ramirez, and J. R. Espinosa, Nature communications **13**, 5717 (2022).
[22] A. R. Tejedor, A. Garaizar, J. Ramírez, and J. R. Espinosa, Biophysical Journal **120**, 5169 (2021).
[23] S. Blazquez, I. Sanchez-Burgos, J. Ramirez, T. Higginbotham, M. M. Conde, R. Collepardo-Guevara, A. R. Tejedor, and J. R. Espinosa, bioRxiv , 2022 (2022).
[24] B. Portz, B. L. Lee, and J. Shorter, Trends in biochemical sciences (2021).
[25] J. Wang, J.-M. Choi, A. S. Holehouse, H. O. Lee, X. Zhang, M. Jahnel, S. Maharana, R. Lemaitre, A. Pozniakovsky, D. Drechsel, *et al.*, Cell **174**, 688 (2018).
[26] S. Maharana, J. Wang, D. K. Papadopoulos, D. Richter, A. Pozniakovsky, I. Poser, M. Bickle, S. Rizk, J. Guillén-Boixet, T. M. Franzmann, *et al.*, Science **360**, 918 (2018).
